# Mechanisms of photoisomerization of the prenylated flavin mononucleotide cofactor: a theoretical study†

**DOI:** 10.1039/d4ra02035a

**Published:** 2024-06-24

**Authors:** Pannipa Panajapo, Phorntep Promma, Kritsana Sagarik

**Affiliations:** a School of Chemistry, Institute of Science, Suranaree University of Technology Nakhon Ratchasima 30000 Thailand kritsana@sut.ac.th +66-44-224635 +66-44-224635

## Abstract

The enzymatic decarboxylation of α,β-unsaturated acids using the ferulic acid decarboxylase (Fdc1) enzyme and prenylated flavin mononucleotide (prFMN) cofactor is a potential, environmentally friendly reaction for the biosynthesis of styrene and its derivatives. However, experiments showed that the enzyme activity of Fdc1 depends on the ring structure of prFMN, namely, the iminium and ketimine forms, and the loss of enzyme activity results from prFMN^im^ → prFMN^ket^ photoisomerization. To obtain insight into this photochemical process and to improve the enzyme efficiency of Fdc1, two proposed photoisomerization mechanisms with different proton sources for the acid–base reaction were studied herein using theoretical methods. The potential energy surfaces calculated using the density functional theory method with the Becke, 3-parameter, and Lee–Yang–Parr hybrid functionals and DZP basis set (DFT/B3LYP/DZP) and TD-DFT/B3LYP/DZP methods confirmed that the light-dependent reaction occurs in the rate-determining proton transfer process and that the mechanism involving intermolecular proton transfer between prFMN^im^ and Glu282 (external base) is energetically more favorable than that involving intramolecular proton transfer in prFMN^im^ (internal base). The thermodynamic results obtained from the transition state theory method suggested that the exothermic relaxation energy in the photo-to-thermal process can promote the spontaneous formation of a high-energy-barrier transition state, and an effective enzymatic decarboxylation could be achieved by slowing down the formation of the undesirable thermodynamically favorable product (prFMN^ket^). Because the rate constant for formation of the high-energy-barrier transition state varies exponentially over the temperature range of 273–298 K, and experimental results have shown that incubating Fdc1 on ice results in a complete loss of enzyme activity, it is recommended to perform the decarboxylation reaction at 285 K to strike a balance between minimizing enzyme stability loss at 273 K and mitigating the effects of UV irradiation. The computational strategy and fundamental insights obtained in this study could serve as guidelines for future theoretical and experimental investigations on the same and similar photochemical systems.

## Abstract 2

The enzymatic decarboxylation of α,β-unsaturated acids using the ferulic acid decarboxylase enzyme and prenylated flavin mononucleotide cofactor is a potential, environmentally friendly reaction for the biosynthesis of styrene and its derivatives.

## Graphical abstract

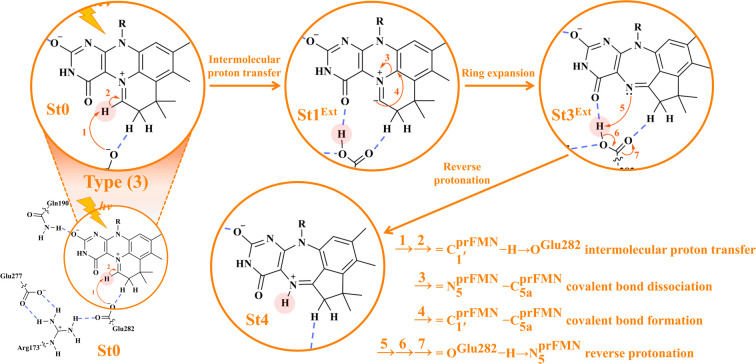

## Introduction

The enzymatic decarboxylation of α,β-unsaturated acids using the ferulic acid decarboxylase (Fdc1) enzyme is envisioned as a potential environmentally friendly process for synthesizing styrene and its derivatives from natural resources.^1–3^ This enzymatic reaction can be effectively accomplished through 1,3-dipolar cycloaddition between substrates and enzyme cofactors,^4^ among which the prenylated flavin mononucleotide (prFMN) has been proven to be effective for the biosynthesis of styrene.^1,3–5,7^ Payne *et al.*^4^ proposed a mechanism for the enzymatic decarboxylation of α,β-unsaturated acids using the prFMN cofactor. The mechanism comprising four consecutive elementary reactions has been widely accepted and further studied using various theoretical and experimental methods. They are 1,3-dipolar cycloaddition, Grob-type decarboxylation, protonation, and retro 1,3-dipolarcycloaddition,^4^ among which the enzyme-catalyzed 1,3-dipolar cycloaddition of prFMN was suggested by a mechanism-based inhibitor experiment to play the most important role.^2^

Ferguson *et al.*^1^ studied the enzyme efficiency of Fdc1 in styrene production from cinnamic acid by monitoring substrate consumption using spectroscopic and kinetic isotope effect methods, from which the cycloelimination was suggested to represent the rate-determining step. In ref. 1, two forms of prFMN with different ring structures were considered, namely, the iminium and ketimine forms, abbreviated prFMN^im^ and prFMN^ket^, respectively. The experimental results indicated that with prFMN^im^, the decarboxylation reaction proceeded *via* 1,3-dipolar cycloaddition, whereas the reaction with prFMN^ket^ occurred *via* Michael addition, and the enzyme activity was confirmed to be higher using prFMN^im^.

To study the effect of the prFMN^im^ and prFMN^ket^ cofactors on the enzyme activity, the enzymatic decarboxylation using Fdc1 to generate styrene from cinnamic acid was further studied.^5^ High-resolution crystal structures and mass spectrometric and kinetic experiments revealed that the prFMN^im^ → prFMN^ket^ isomerization could occur through an irreversible photochemical reaction, which is independent of the Glu277–Arg173–Glu282 residue network. This photoisomerization reaction was suggested to be the key factor for the loss of Fdc1 enzyme activity.

**Fig. 1:**
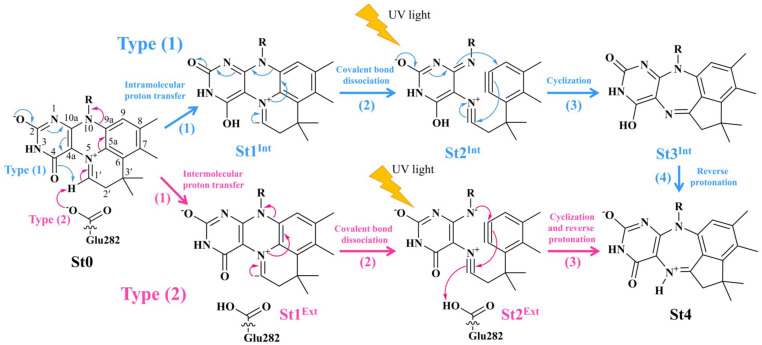
The proposed prFMN^im^ → prFMN^ket^ photoisomerization mechanisms obtained based on high-resolution crystal structures, mass spectrometric and kinetic experiments.^5^ All the symbols are explained in the text. Type (1) = mechanism involving intramolecular proton transfer in the 
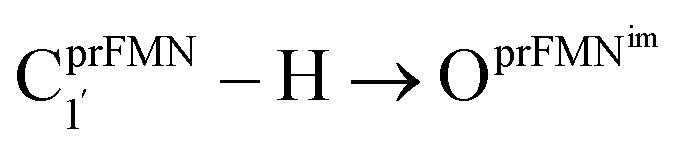
 H-bond in prFMN^im^ with four consecutive elementary reactions; Type (2) = mechanism involving intermolecular proton transfer in the 
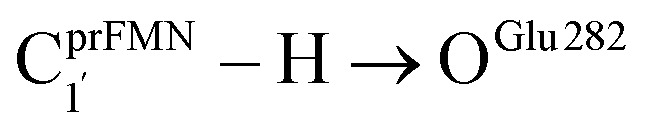
 H-bond between prFMN^im^ and Glu282 with three consecutive elementary reactions. Int = internal base; Ext = external base.

Two photoisomerization mechanisms with different proton sources for the acid–base reaction are proposed in Fig. 1.^5^ Type (1) mechanism involves four consecutive elementary reactions and begins with the intramolecular proton transfer in prFMN^im^, whereas for Type (2) mechanism, the intermolecular proton transfer between prFMN^im^ and Glu282 represents the initial process with only three consecutive elementary reactions. Although the elementary reactions in Type (1) and (2) mechanisms were not known in detail, the light-dependent reaction was anticipated to be in the cyclization process, in which the exposure to UV light at *λ*^abs^ = 365 nm for 5 min led to a complete loss of enzyme activity and change in the absorption spectrum.^5^ This light-dependent reaction is similar to maleimide [5 + 2] photocycloaddition reported in ref. 6.

In our previous study,^7^ the elementary reactions proposed in ref. 4 were examined in low and high local dielectric environments (*ε* = 1 and 78) using the density functional theory (DFT) method with the Becke, 3-parameter, and Lee–Yang–Parr hybrid functionals and DZP basis set (DFT/B3LYP/DZP) and the transition state theory (TST) method. The active site models included the α-methylcinnamate (Cin) substrate, prFMN^im^ cofactor, and all relevant Fdc1 residues. The results confirmed that the Fdc1 backbone does not play an important role in the decarboxylation reaction and that indirect cycloelimination in a low local dielectric environment is the rate-determining step.

Literature review showed that in the past decade (2014–2024), there were 44 published research articles reporting the design and/or improvement of enzymatic decarboxylation in organic syntheses, among which 30 studies focused on the decarboxylation of α,β-unsaturated acids, and 14 studies used the Fdc1 enzyme in the biosynthesis of styrene and its derivatives. Literature review also revealed that only 5 studies investigated the photoisomerization of the enzyme cofactors under UV exposure, among which only one experimental study^5^ examined in detail the loss of the enzyme activity through the prFMN^im^ → prFMN^ket^ photoisomerization.

Because there is no theoretical study on this photoisomerization reaction, to bridge the gap between the experimental and theoretical knowledge, Type (1) and Type (2) mechanisms were studied in detail using the DFT/B3LYP/DZP, TD-DFT/B3LYP/DZP, and TST methods. To obtain insight into the prFMN^im^ → prFMN^ket^ photoisomerization, this mechanistic study focused on the molecular processes (scenarios) in the lowest singlet excited (S_1_) state and on the kinetics and thermodynamics of nonradiative reaction pathways. The theoretical investigation began with calculations of the equilibrium structures and spectroscopic properties of the selected active site models, from which the potential energy surfaces (PESs) for prFMN^im^ → prFMN^ket^ were optimized in the S_1_ and S_0_ states. The kinetic and thermodynamic properties of the PESs were computed using the TST method. To suppress/delay the prFMN^im^ → prFMN^ket^ photoisomerization process, appropriate thermodynamic conditions were suggested in order to improve the enzymatic decarboxylation of α,β-unsaturated acids using Fdc1.

## Computational methods

### Active site models

**Fig. 2:**
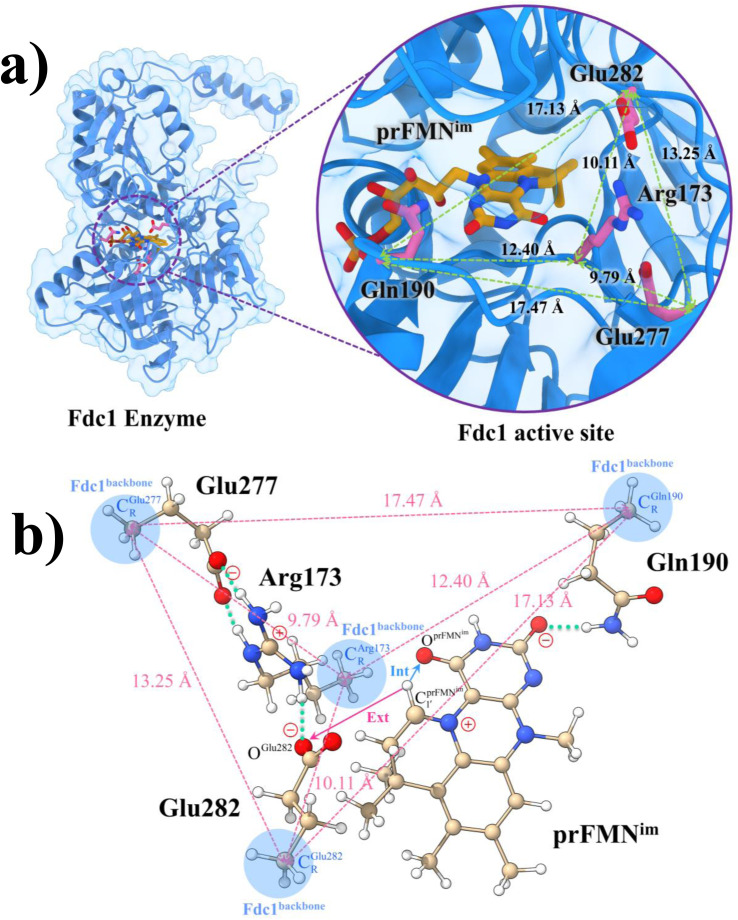
(a) The structures of the active site, precursor, cofactor and residues involved in the enzymatic decarboxylation of α,β-unsaturated acid using Fdc1 enzyme and prFMN^im^ cofactor obtained from DFT/B3LYP/DZP geometry optimizations.^7^ (b) An example of the model active site cluster (St0) used in the study of the prFMN^im^ → prFMN^ket^ photoisomerization. Dash lines show the residue network distances. Int = intramolecular proton transfer in the 
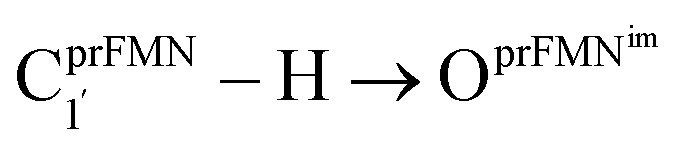
 H-bond of prFMN^im^; Ext = intermolecular proton transfer in the 
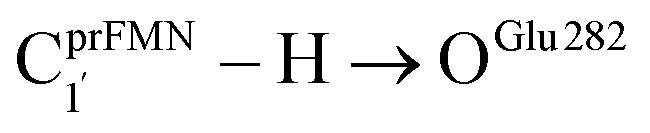
 H-bond between prFMN^im^ and Glu282; C^Arg173^_R_, C^Gln190^_R_, C^Glu277^_R_ and C^Glu282^_R_ = carbon atoms of the CH_3_ groups that substitute the carbon atoms of the Fdc1 backbone.

Because the analysis of the equilibrium structures and PESs in our previous study^7^ showed that the Fdc1 backbone (Fig. 2a) does not play an important role in the decarboxylation reaction, the active site was modeled in this work by substituting the carbon atoms of the Fdc1 backbone that connect the residues with CH_3_ groups (Fig. 2b). For example, C^Gln190^_R_ is the carbon atom of the CH_3_ group that substitutes the carbon atom of the Fdc1 backbone that connects the Gln190 residue. Although the experiment suggested that prFMN^im^ → prFMN^ket^ photoisomerization is independent of the Glu277–Arg173–Glu282 residue network,^5^ to obtain a realistic picture, the active site models used in this study consisted of Glu277, Arg173, Glu282, and Gln190 and prFMN^im^ (Fig. 2b), regarded as the “active site cluster.”

### Equilibrium structures

**Fig. 3:**
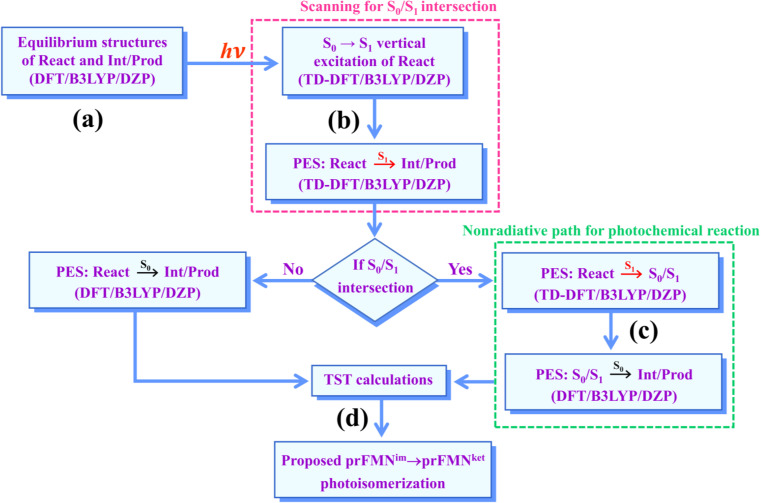
The computational strategy and methods used to study the prFMN^im^ → prFMN^ket^ photoisomerization. DFT/B3LYP/DZP = density functional theory with the B3LYP functional and DZP basis set; TD-DFT/B3LYP/DZP = time-dependent density functional theory with the B3LYP functional and DZP basis set; PES = potential energy surface; TST = transition state theory; (S_0_/S_1_) = intersection of the S_0_ and S_1_ states; react, Int/prod = reactant, intermediate and product, respectively.

The computational strategy and methods used in this study are shown in Fig. 3. The equilibrium structures of prFMN^im^, prFMN^ket^, Glu277, Arg173, Glu282, and Gln190 (Table S1†) were optimized primarily in the S_0_ state (step (a) in Fig. 3) using the DFT/B3LYP/DZP method and the S_0_ → S_1_ energies (Δ*E*^Ex^) were calculated using the TD-DFT/B3LYP/DZP method. These computational methods were chosen based on our good experience and benchmark calculations in several mechanistic studies. For example, for the photodissociation and formation of glycine,^8,9^ our benchmark calculations against the complete active space multiconfigurational second-order perturbation theory (CASPT2) method showed that the characteristic structures and energies on the S_0_ and S_1_ PESs (*e.g.*, the transition structures and structures at the S_0_/S_1_ intersection) obtained from the DFT/B3LYP and TD-DFT/B3LYP methods were comparable with those obtained from the CASPT2 method, and the DFT/B3LYP/DZP and DFT/B3LYP/TZP methods yielded approximately the same equilibrium structures, relative interaction energies and energy barriers on the PESs for bifunctional proton transfers in the hydrogen-bonds (H-bonds) in poly(benzimidazole) (PBI) systems.^10^

In this work, although our model active site clusters contain aromatic compounds, the dispersion correction was not included in the DFT and TD-DFT calculations, because the predominant intermolecular interactions are the H-bond and electrostatic interactions, and there is no π–π interaction directly involved on the hypothesized reaction pathways. Because the model active site clusters used in this work are large in view of quantum chemical methods, based on which the energy gradients on the S_0_ and S_1_ PESs had to be repeatedly calculated in the reaction pathway optimizations, to compromise between the numerical accuracy and computational resources, we decided to use the DFT/B3LYP/DZP and TD-DFT/B3LYP/DZP methods without the dispersion correction.

Remarks should be made on the application of the TD-DFT method with the B3LYP hybrid functional. The TD-DFT method has been a predominant method for excited state calculations on large molecules. The TD-DFT method circumvents explicit state calculations by focusing only on excitation energy and transition dipole moment.^11^ However, benchmark TD-DFT calculations revealed that pure density functionals tend to underestimate transition energies, whereas hybrid functionals such as B3LYP yield better results especially for low-lying singlet transition states.^12^

Practically, the presence of nearly degenerate HOMO and LUMO energies, particularly at the intersections of the S_0_ and S_1_ states, introduces spin instability, which is associated with holes positioned below the Fermi level. The theoretical results reported in ref. 13 demonstrated that for the photochemical ring opening in oxirane, this problem can be avoided by employing the Tamm–Dancoff approximation (TDA) in conjunction with the TD-DFT method. Because the TD-DFT/TDA method is a simplification of the full TD-DFT method, in which the full TD-DFT equations are truncated to mitigate the algorithmic failure due to spin instability, the computational efficiency can be significantly increased.^14^ Therefore, the TD-DFT/B3LYP method with TDA was applied in the present and all of our previous studies on large molecular systems.^15,16^

Analysis of the elementary reactions in Type (1) and (2) mechanisms^5^ resulted in seven active site clusters. In the initial active site cluster St0 (Fig. 1), prFMN^im^ is connected to Gln190*via* the N^Gln190^–H⋯O^prFMNim,−^ H-bond (Fig. 2b). The Glu277–Arg173–Glu282 network is associated *via* a carboxylate–guanidinium–carboxylate (COO^−^⋯Gdm^+^⋯COO^−^) salt-bridge-type interaction (Fig. 2b), which partly stabilizes the molecules in the active site clusters. The equilibrium structures and Δ*E*^Ex^ of the active site clusters were calculated using the DFT/B3LYP/DZP and TD-DFT/B3LYP/DZP methods, respectively (Table S2†). All quantum chemical calculations were performed using the TURBOMOLE 7.50 software package.^17^ The absorption spectra of the active site clusters were computed from 500 Wigner sampled structures using the NEWTON-X software package^18^ interfaced with TURBOMOLE 7.50.

### prFMN^im^ → prFMN^ket^ photoisomerization pathways

To simplify the discussion, the symbols shown in Fig. 1 are used and the proposed photoisomerization pathways^5^ are explained in detail. Type (1) mechanism comprises four elementary reactions: (1) 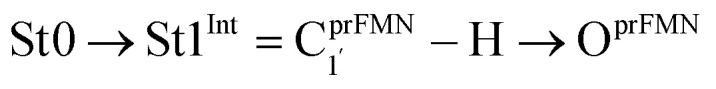 intramolecular proton transfer; (2) St1^Int^ → St2^Int^ = covalent bond dissociation; (3) St2^Int^ → St3^Int^ = cyclization; and (4) St3^Int^ → St4 = reverse protonation. For the Type (2) mechanism, only three elementary reactions were proposed: (1) 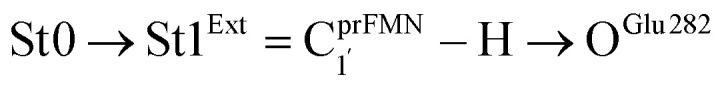 intermolecular proton transfer; (2) St1^Ext^ → St2^Ext^ = covalent bond dissociation; and (3) St2^Ext^ → St3^Ext^ → St4 = concerted cyclization and reverse protonation.

To characterize the structures and energetics of the active site clusters on the PESs, additional symbols are used; […]^Int^ and […]^Int,*^ denote structures on the S_0_ and S_1_ PESs of the Type (1) mechanism, respectively; […]^Ext,‡^ and […]^Ext,§^ denote transition structure (‡) and structure at the S_0_/S_1_ intersection (§) on the PESs of the Type (2) mechanism, respectively; Δ*E*^Ex^ = S_0_ → S_1_ energy; Δ*E*^§^ = energy at the S_0_/S_1_ intersection (§); and Δ*E*^‡^ = energy barrier. The equilibrium structures of the active site clusters obtained from the DFT/B3LYP/DZP geometry optimization (Table S2†) were used in the elementary reaction pathway optimization in the S_1_ state (step (b) in Fig. 3). The elementary reaction pathway optimization was aimed at finding the possibility of the S_1_ → S_0_ nonradiative relaxation of the S_0_ → S_1_ excited active site clusters.

Because the light-induced reaction was not clearly identified in ref. 5, the S_0_ → S_1_ excited precursors for photoisomerization were hypothesized based on the radiation wavelength used in the experiment (*λ*^abs^ = 365 nm/Δ*E*^Ex^ = 3.40 eV), Δ*E*^Ex^ obtained from TD-DFT/B3LYP/DZP calculations, and Δ*E*^‡^ for the formation of the precursors in the S_0_ state. Herein, 7–15 structures connecting the precursor, transition state, and intermediate/product were optimized using a constrained optimization algorithm^19^ included in TURBOMOLE 7.50. If the S_0_/S_1_ intersection was found, the pathway being optimized was refined using the S_0_/S_1_ structure as the intermediate/product (step (c) in Fig. 3).

### Kinetics and thermodynamics of photoisomerization

Characteristic structures of the model active site clusters on the S_0_ and S_1_ PESs were used for the calculation of the rate constants using the TST method (step (d) in Fig. 3).^20,21^ Herein, the quantized-vibrational rate constants (*k*^Q-vib^) were computed over the temperature range of 273–353 K using eqn (1):^22^1
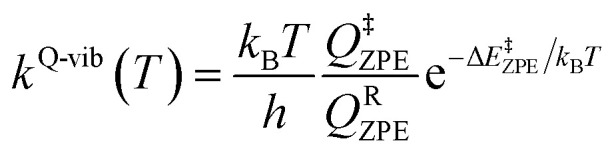


In eqn (1), the energy barrier (Δ*E*^‡^_ZPE_) was obtained with the zero-point vibrational energy correction. *Q*^R^_ZPE_ and *Q*^‡^_ZPE_ are the partition functions of the precursor and transition structures, respectively. *k*_B_ and *h* are the Boltzmann and Planck constants, respectively.

The thermodynamic properties of interest were the activation free energies (Δ*G*^‡^) and enthalpies (Δ*H*^‡^). They were derived from *k*^Q-vib^ (*T*) using eqn (2) and (3), respectively:2
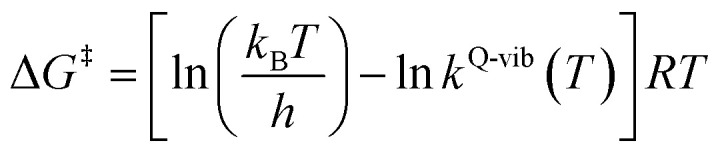
3
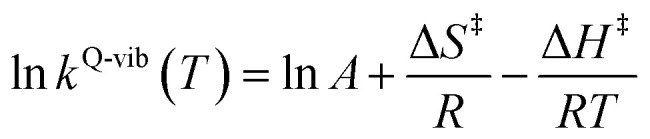


Δ*S*^‡^ in eqn (3) is the activation entropy and *R* is the gas constant. The value of Δ*H*^‡^ is obtained from the slope of the linear relationship between ln *k*^Q-vib^ (*T*) and 1000/*T*. To calculate the thermodynamic properties of the nonradiative photoisomerization pathway, the S_0_ → S_1_ excited active site clusters were assumed to barrierlessly relax to the S_0_/S_1_ intersection and to the structures in the S_0_ state.

**Fig. 4:**
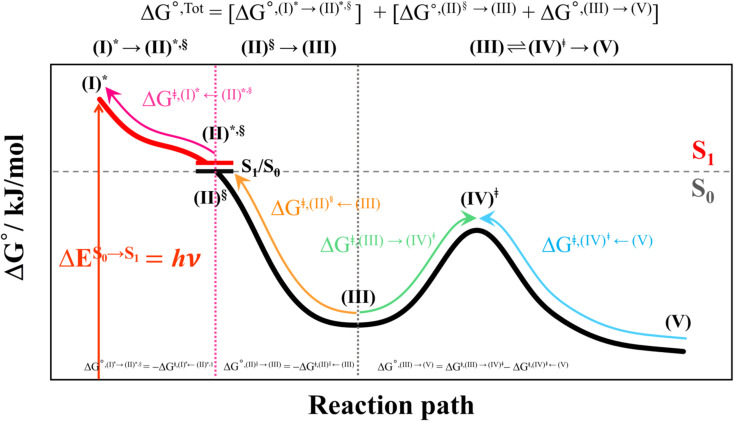
Schematic diagram showing calculations of Gibbs free energies on the proposed “photo-to-thermal pathway” using the TST method. All the symbol used are explained in the kinetics and thermodynamics of photoisomerization subsection. (I)^*^ → (II)^*,§^ = relaxation of the S_0_ → S_1_ vertically excited structure (I)^*^ to structure (II)^*,§^ at the S_0_/S_1_ intersection; (II)^§^ → (III) ⇌ (IV)^‡^ → (V) = relaxation of structure (II)^§^ at the S_0_/S_1_ intersection to the equilibrium structure (V) in the S_0_ state.

This led to the consecutive “photo-to-thermal pathway” in Fig. 4, the (I)^*^ → (II)^*,§^ relaxation in the S_1_ state followed by (II)^§^ → (III) ⇌ (IV)^‡^ → (V) in the S_0_ state, in which (III) ⇌ (IV)^‡^ → (V) was assumed to be in quasi-equilibrium. Based on this hypothesized consecutive reaction pathway, the total Gibbs free energy (Δ*G*°^,Tot^) was computed using Δ*G*^‡^ obtained from the TST method. For (I)^*^ → (II)^*^, Δ*G*°^,(I)*→(II)*,§^ = −Δ*G*_r_^‡,(I)*←(II)*,§^, whereas Δ*G*°^,(I)*→(III)^ = −(Δ*G*_r_^‡,(I)*←(II)*,§^ + Δ*G*_r_^‡,(II)§←(III)^) for (I)^*^ → (II)^*,§^/(II)^§^ → (III). For (III) ⇌ (IV)^‡^ → (V), Δ*G*°^,(III)→(V)^ = Δ*G*_f_^‡,(III)→(IV)‡^ − Δ*G*_r_^‡,(IV)‡←(V)^. Therefore, Δ*G*°^,Tot^ = Δ*G*°^,(I)*→(III)^ + Δ*G*°^,(III)→(V)^. Likewise, the Gibbs free energy for the formation of the transition structure (IV)^‡^ in the S_0_ state Δ*G*°^,(I)*→(IV)‡^ = Δ*G*°^,(I)*→(III)^ + Δ*G*_f_^‡,(III)→(IV)‡^. The same method was used to calculate the entropy changes (Δ*S*°^,Rx^) of the isomerization reaction (system). All the kinetic and thermodynamic properties were calculated using the DL-FIND program^23^ included in the ChemShell software package.^24^

## Results and discussion

### Equilibrium structures of the active site clusters

The DFT/B3LYP/DZP results show that starting from the seven hypothesized active site clusters shown in Fig. 1, the optimized structures are slightly changed (Table S2a†), except for St2^Int^ and St2^Ext^. For St2^Int^, DFT/B3LYP/DZP geometry optimization yielded the precursor St0 (prFMN^im^ in the active site cluster), whereas starting from St2^Ext^ resulted in the product St4 (prFMN^ket^ in the active site cluster). These results suggest that only five active site clusters are stationary points (intermediates/products) on the S_0_ PESs, and St2^Int^ and St2^Ext^ could only be the transition structures on the reaction pathways.

Analysis of the equilibrium structures of the five active site clusters revealed that in the S_0_ state, the residue-to-residue (R-to-R) distances in Fig. 2b are not significantly different, characterized by standard deviations less than ±0.15 Å (Table S2b†); the average R-to-R distances were calculated using the distances between the carbon atoms of the CH_3_ groups that substituted the carbon atom of the Fdc1 backbone. For example, 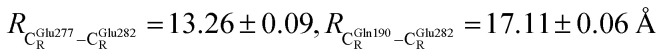, and 
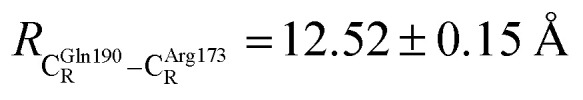
. Because our previous study^7^ also revealed that the residue-to-residue distances (active site volume) do not change significantly during the enzymatic reaction, the lock-and-key model could explain the substrate specificity of the Fdc1 enzyme, and the catalytic efficiency of this enzymatic decarboxylation reaction is partly connected to the efficiency of the proton exchange between the substrate and Glu282, which is a part of the conserved Glu277–Arg173–Glu282 residue network. These results further suggested that the model active site clusters without the Fdc1 backbone are reasonable and can be used for further studies.

DFT/B3LYP/DZP geometry optimization further suggested that St4 possesses the lowest total energy in the S_0_ state, and St0 is 24 kJ mol^−1^ less stable. The TD-DFT/B3LYP/DZP method showed that the S_0_ → S_1_ energies of prFMN^im^ and prFMN^ket^ are Δ*E*^Ex^ = 3.15 and 2.25 eV (*λ*^S_0_→S_1_^ = 394 and 551 nm), respectively. Comparison of Δ*E*^Ex^ in Tables S1 and S2a† indicated that Δ*E*^Ex^ of St0 is red-shifted from that of prFMN^im^; Δ*E*^Ex^ of St0 is 0.55 eV (∼83 nm red-shifted) lower than that of prFMN^im^, whereas Δ*E*^Ex^ of St4 and that of prFMN^ket^ are almost the same. The red-shift is hypothesized to result from strong H-bond interaction between prFMN^im^ and residues in the S_1_ state; based on TD-DFT calculations on the camphorsulfonic acid doped polyaniline,^25^ the electronic spectral red- and blue-shifts could be induced by the excited state H-bond dynamics, for which strong H-bond interaction in the excited states leads to an increase in the oscillator strength and red-shift.

**Fig. 5:**
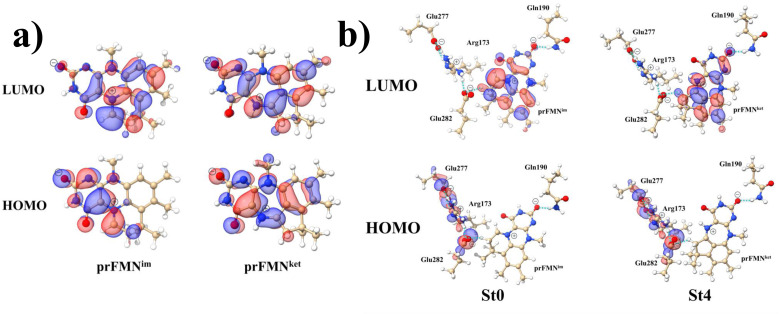
The HOMOs and LUMOs of the prFMN^im^ and prFMN^ket^ cofactors (a), and model active site clusters (b), obtained from DFT/B3LYP/DZP geometry optimizations.

Analysis of the HOMOs and LUMOs in Fig. 5a suggested that while the electron density distributions for prFMN^ket^ in the S_0_ and S_1_ states are not substantially different, those for prFMN^im^ are significantly different, especially for the HOMOs, which are characterized by a lower π-character in the phenyl ring. The H-bond formation between the cofactor and residues results in nearly the same electron density distributions in the S_0_ and S_1_ states for St0 and St4 (Fig. 5b); in the S_0_ state, electron density distributions for St0 and St4 are extensive in the Glu277–Arg173–Glu282 network, whereas in the S_1_ state, the electron densities at the cofactor are the highest, resulting in an increase in the π-character at the cofactors. This could explain why Δ*E*^Ex^ for St0 and St4 are not significantly different (Table S2a†), Δ*E*^Ex^ = 2.60 and 2.69 eV, respectively.

Remarks should be made on the HOMOs of the Glu277–Arg173–Glu282 network in Fig. 5b. The extensive charge (electron density) distributions in the S_0_ state within the Glu277–Arg173–Glu282 network reflect the predominant electrostatic interactions in the COO^−^⋯Gdm^+^⋯COO^−^ salt-bridge; Gdm^+^ stabilizes the two carboxylate anions. Because similar COO^−^⋯Gdm^+^⋯COO^−^ salt-bridges were found in many protein structures, and theoretical studies have shown that the Gdm^+^⋯COO^−^ attractive interaction affects the ligand recognition and binding, as well as enzyme folding and activity, the COO^−^⋯Gdm^+^⋯COO^−^ salt-bridge with specific electrostatic interaction could be synthesized and applied, for example, in drug design; Gdm^+^ derivatives have already been used to treat various deceases related to muscle weakness, cancer and diabetes.^26^

### Potential energy surfaces for prFMN^im^ → prFMN^ket^ photoisomerization

The structures and energies of the active site clusters on the S_0_ and S_1_ PESs obtained from the DFT/B3LYP/DZP and TD-DFT/B3LYP/DZP reaction path optimization (Fig. S1–S6†) are analyzed in detail. To study the possibility of St2^Int^ and St2^Ext^ being the S_0_ → S_1_ photoexcited precursors in the [5 + 2] photocycloaddition, as proposed in ref. 6, the PESs for St0 → St1^Int^ → St2^Int^ and St0 → St1^Ext^ → St2^Ext^ in the S_0_ state were primarily calculated; the intra- and intermolecular proton transfers and covalent bond dissociation occurring in the S_0_ state serve as prerequisites for the light-dependent cycloaddition in Type (1) and Type (2) mechanisms,^5^ respectively.

The results showed that both St0 → St1^Int^ → St2^Int^ and St0 → St1^Ext^ → St2^Ext^ involve extraordinarily high energy barriers in the S_0_ state, especially for the covalent bond dissociation, Δ*E*^‡^ = 456.4 and 435.9 kJ mol^−1^ (Fig. S1b and S2b,† respectively). Based on these findings and the observations that St2^Int^ and St2^Ext^ are not stationary points with S_0_ → S_1_ energies significantly different from the experimental radiation wavelength (*λ*^abs^ = 365 nm/Δ*E*^Ex^ = 3.40 eV),^5^ these two active site clusters were ruled out from further study; TD-DFT/B3LYP/DZP single point calculations suggested that for St2^Int^ and St2^Ext^, Δ*E*^Ex^ = 2.96 and 1.88 eV, respectively (Table S2a†).

**Fig. 6:**
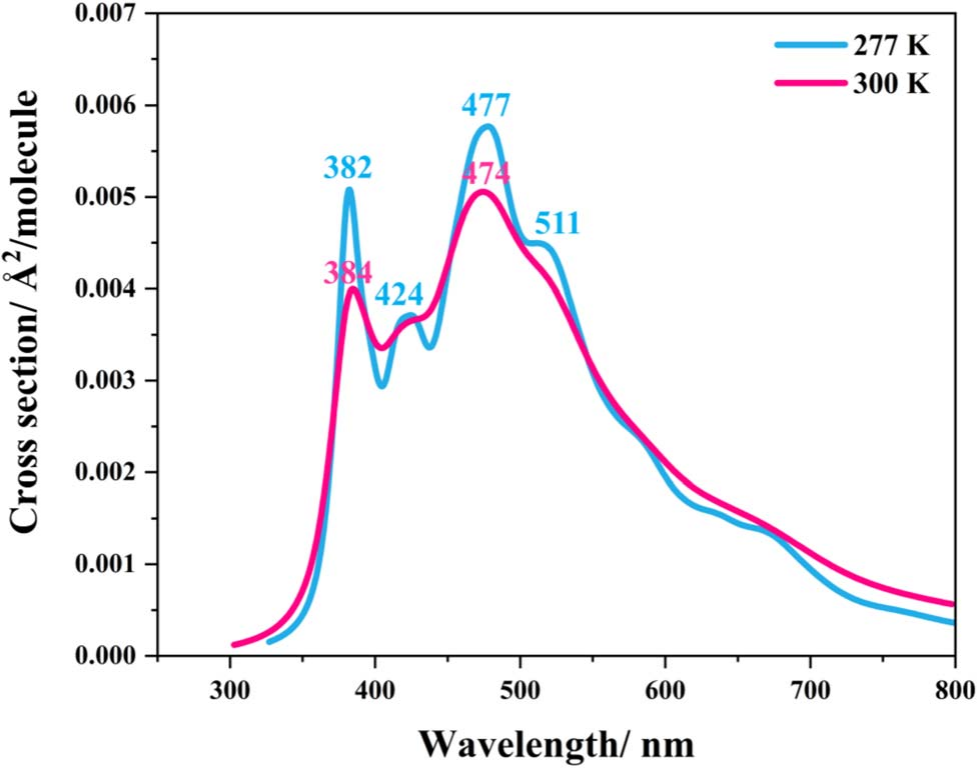
The UV-visible spectra of the model active site cluster St0 (prFMN^im^ in the residue network) obtained based on 500 Wigner sample structures at 277 and 300 K.

Similarly, because the intra- and intermolecular proton transfers (St0 → St1^Int^ and St0 → St1^Ext^) possess high energy barriers in the S_0_ state, Δ*E*^‡^ = 262.6 (Fig. S1a†) and 125.7 kJ mol^−1^ (Fig. S2a†), respectively, it is unlikely that St1^Int^ and St1^Ext^ serve as the photoexcited precursors for S_0_ → S_1_. To study the possibility of St0 being the S_0_ → S_1_ photoexcited precursor, UV-visible absorption spectra were calculated at 277 and 300 K using 500 Wigner sampled initial conditions. The results in Fig. 6 show two outstanding peaks, *e.g.*, at 300 K, *λ*^abs^ = 384 and 474 nm. Because the structured peak at *λ*^abs^ = 384 nm is close to the radiation wavelength used in an experiment,^5^ St0 was chosen as the S_0_ → S_1_ photoexcited precursor for Type (1) and Type (2) mechanisms.

The PESs obtained from reaction path optimization show that the S_0_ → S_1_ vertically excited structure barrierlessly relaxes to the structure at the S_0_/S_1_ intersection (St0^*^ → St0^*,§^ in Fig. S3a†). The S_0_ PES (Fig. S4†) illustrates that after the S_1_ → S_0_ nonradiative relaxation, the intramolecular proton transfer (St0^§^ → St0-[1]^Int^ → St1^Int^) in the Type (1) mechanism possesses a rather high energy barrier, Δ*E*^‡^ = 209.5 kJ mol^−1^, whereas Δ*E*^‡^ = 86.9 kJ mol^−1^ is for the intermolecular proton transfer (St0^§^ → St0-[1]^Ext^ → St1^Ext^) in the Type (2) mechanism (Fig. S5a†). Hence, one can conclude that the external base pathway is energetically more favorable than the internal one, and only the Type (2) mechanism is further discussed in detail.

To test the hypothesis that the red-shift in Δ*E*^Ex^ for St0 compared to that of prFMN^im^ results from strong H-bond interaction between the prFMN^im^ cofactor and residues in the S_1_ state, the variation of the N^Gln190^–H⋯O^prFMN,−^ H-bond distance in the S_1_ state was analyzed as an example. The results revealed that on the S_1_ PES (Fig. S3a†), while the *R*_N^Gln190^–H⋯O^prFMN,−^_ H-bond distance decreases, the *R*_N^Gln190^–H_ distance increases (Fig. S3b†), reflecting an increase in the N^Gln190^–H⋯O^prFMN,−^ H-bond strength (red-shift) in the S_1_ state towards the S_0_/S_1_ intersection.

**Fig. 7:**
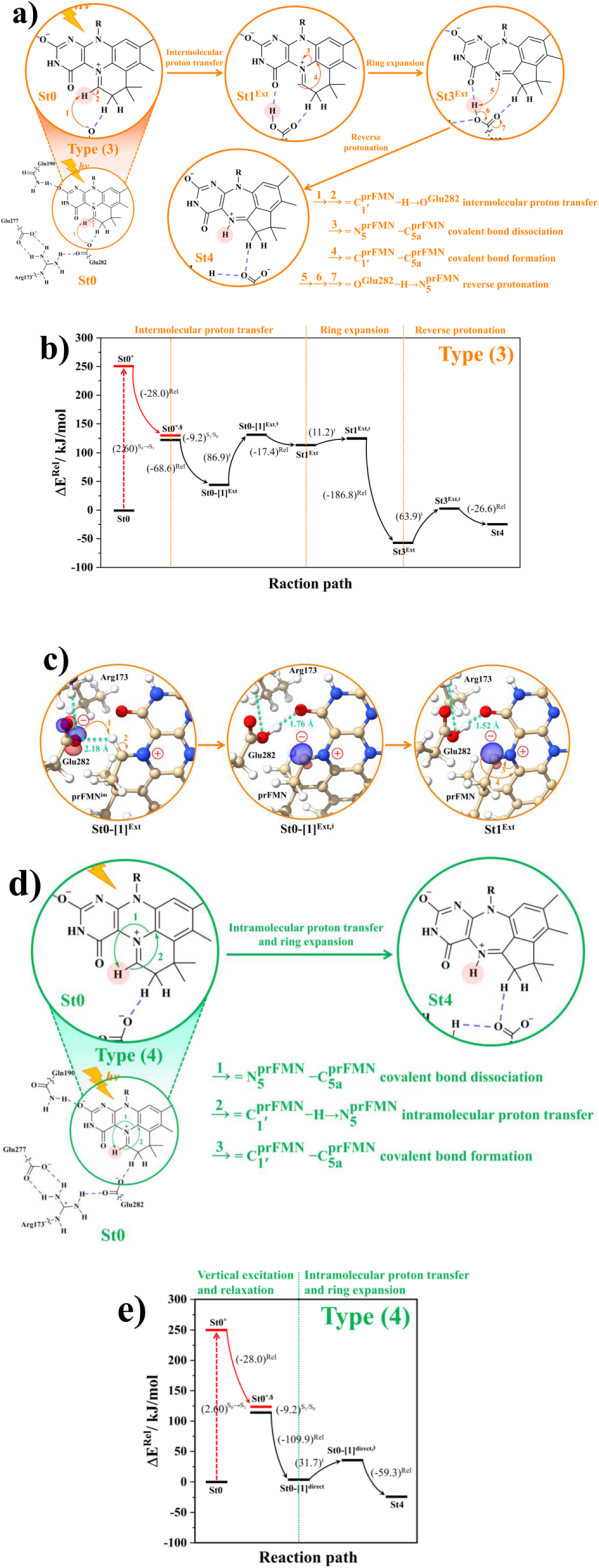
(a) and (b) Type (3) mechanism and potential energy profiles involving the S_0_ → S_1_ excitation of St0, intermolecular proton transfer in 
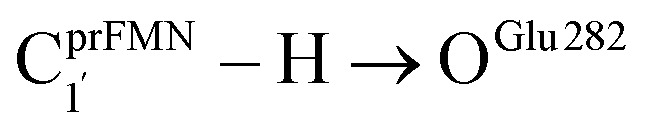
 H-bond between prFMN^im^ and Glu282, ring expansion and reverse protonation, respectively. (c) The H-bond distances (
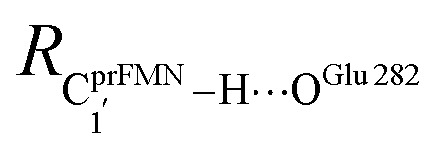
 and R__O_^Glu282^–H⋯O^prFMN^_) and local valence charge densities on the high-energy barrier intermolecular proton transfer process (St0-[1]^Ext^ → St0-[1]^Ext,‡^ → St1^Ext^). (d) and (e) Type (4) mechanism and potential energy profiles involving the S_0_ → S_1_ excitation of St0 and concerted 
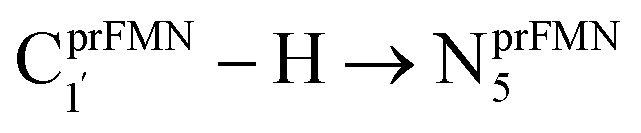
 intramolecular proton transfer in prFMN^im^ and ring expansion. The characteristic structures are shown in detail in Table S2.† (…)^S_0_ → S_1_^ = S_0_ → S_1_ vertical excitation energy; (…)^Rel^ and (…)^‡^ = relative and transition energies on the PES; (…)^S_1/_S_0_^ = difference between the total energies in the S_0_ and S_1_ states at the S_0_/S_1_ intersection.

Because St2^Ext^ had already been excluded from the photoisomerization pathway, an attempt was made to bypass this active site cluster. The results showed that after the S_1_ → S_0_ nonradiative relaxation at the S_0_/S_1_ intersection (Fig. S3a†) and 
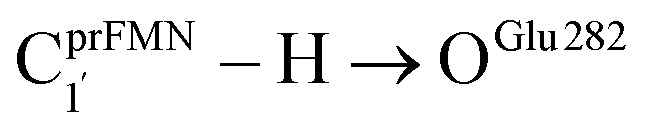
 intermolecular proton transfer from prFMN^im^ to Glu282 (Fig. S5a†), the S_0_ PES shows a low energy barrier for St1^Ext^ → St3^Ext^, Δ*E*^‡^ = 11.2 kJ mol^−1^ (Fig. S5b†), represented by concerted N^prFMN^_5_–C^prFMN^_5a_ and 
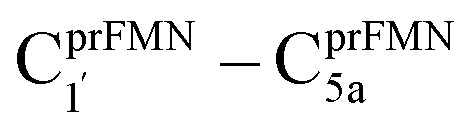
 covalent bond dissociation and formation in Fig. 7a, respectively. This concerted process can be collectively regarded as “ring expansion.” To complete the formation of the St4 product (prFMN^ket^ in the active site cluster), the S_0_ PES for the reverse protonation from Glu282 to prFMN^im^ (St3^Ext^ → St4) has Δ*E*^‡^ = 63.9 kJ mol^−1^ (Fig. S5c†). The potential energy profile, which bypasses St2^Ext^, is regarded as the Type (3) mechanism shown in Fig. 7b.

The analysis of the H-bond distances, local valence charge densities and energetics in the high-energy barrier intermolecular proton transfer process (St0-[1]^Ext^ → St0-[1]^Ext,‡^ → St1^Ext^) in Fig. 7c reveals that although the 
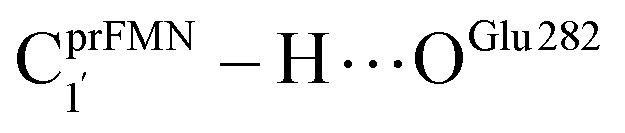
 H-bond in St0-[1]^Ext^
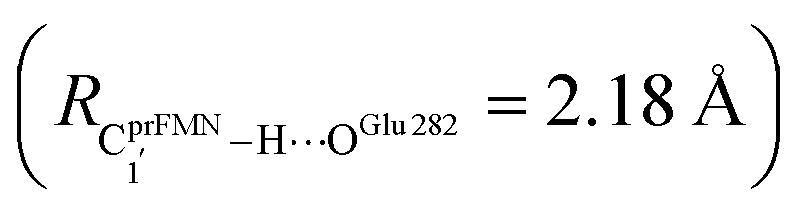
 is longer than the O^Glu282^–H⋯O^prFMN^ H-bond in St1^Ext^ (*R*_O^Glu282^–H⋯O^prFMN^_ = 1.52 Å), due to the strong electrostatic interaction between Glu282 and prFMN^im^, St0-[1]^Ext^ is more stable than St1^Ext^. It also appears that proton transfer to one of the COO^−^ groups in the COO^−^⋯Gdm^+^⋯COO^−^ salt bridge leads to a strong decrease in the electrostatic interaction between Glu282 and prFMN^im^, recognized from a reduction of the local valence charge density at the COOH group of St1^Ext^, and the high-energy barrier in St0-[1]^Ext^ → St0-[1]^Ext,‡^ → St1^Ext^ could be attributed the 
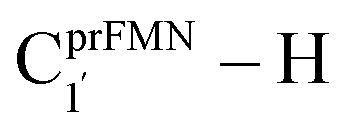
 covalent bond breaking.

Because the Type (1) mechanism^5^ has been established in this work to be energetically not favorable, to search for an alternative internal base pathway, the S_0_ PES directly connecting structure St0^§^ at the S_0_/S_1_ intersection and St4 (St0^§^ → St4) was optimized. It appeared that St0^§^ → St0-[1]^direct^ → St0-[1]^direct,‡^ → St4 (Fig. S6†) can occur without proton exchange between the prFMN^im^ cofactor and the Glu282 residue. This internal base pathway (Type (4) mechanism shown in Fig. 7d) involves concerted 
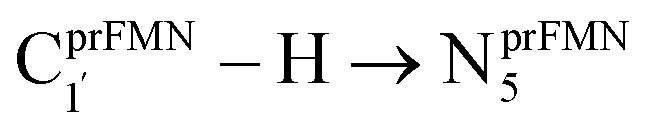
 intramolecular proton transfer, N^prFMN^_5_–C_5a_^prFMN^ and 
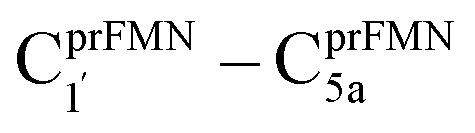
 covalent bond dissociation and formation, respectively, with Δ*E*^‡^ = 31.7 kJ mol^−1^ (Fig. 7e).

### Thermodynamics of the elementary reactions

To study the kinetic and thermodynamic properties of the two energetically favorable pathways, the characteristic active site clusters in Type (3) and Type (4) mechanisms shown in Fig. 7 were used in TST calculations, and the results are summarized in Tables S3–S5.† The results discussed are included in Tables 1 and 2. Analysis based on the photo-to-thermal pathway in Fig. 4, (I)^*^ → (II)^*,§^/(II)^§^ → (III), revealed that for the Type (3) mechanism at *T* = 298 K, although the entropy for the formation of St0-[1]^Ext,‡^ is negative (Δ*S*°^,(I)*→(IV)‡^ = −7.4 × 10^−2^ kJ mol^−1^), the high exothermic relaxation energy for St0^*^ → St0^§^ → St0-[1]^Ext^ (Δ*H*°^,(I)*→(III)^ = −178.4 kJ mol^−1^ in Table 1a) allows the spontaneous formation of this high energy-barrier transition state with Δ*G*°^,(I)*→(IV)‡^ = −77.7 kJ mol^−1^.

**Table 1:** Thermodynamics of the elementary reactions for the external base prFMN^im^ → prFMN^ket^ photoisomerization (Type (3) mechanism), obtained from the DFT/B3LYP/DZP, TD-DFT/B3LYP/DZP and TST methods. Δ*G*° and Δ*H*° = Gibbs free energy and enthalpy in kJ mol^−1^; Δ*S*° = entropy in kJ mol^−1^ K^−1^; Δ*G*°^,Tot^ = total reaction Gibbs free energy for prFMN^im^ → prFMN^ket^ and; Δ*S*°^,Rx^ = reaction entropy of all the elementary processes; *T* = temperature in K

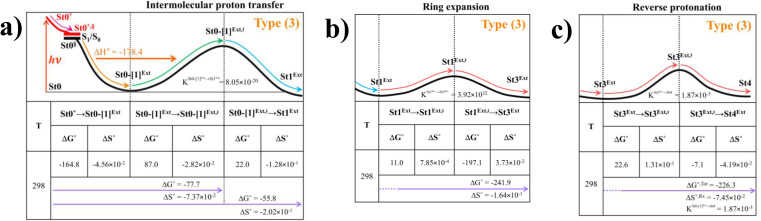

Based on the same approach, the total Gibbs free energy and reaction entropy for the prFMN^im^ → prFMN^ket^ photoisomerization *via* the Type (3) mechanism were computed to be ΔG°^,Tot^ = −226.3 kJ mol^−1^ and ΔS°^,Rx^ = −7.5 × 10^−2^ kJ mol^−1^ K^−1^ at 298 K (Table 1c). For the Type (4) mechanism, Δ*H*°^,(I)*→(III)^ = −221.1, Δ*G*°^,Tot^ = −226.3 kJ mol^−1^, and Δ*S*°^,Rx^ = −7.4 × 10^−2^ kJ mol^−1^ K^−1^, with the spontaneous formation of St0-[1]^direct,‡^, Δ*G*°^,(I)*→(IV)‡^ = −131.6 kJ mol^−1^ (Table 2). Therefore, based on the values of Δ*G*°^,(I)*→(IV)‡^ of the rate-determining step, the Type (4) mechanism is thermodynamically more favorable than the Type (3) mechanism. Because Δ*G*°^,Tot^ and Δ*S*°^,Rx^ of Type (3) and Type (4) mechanisms are the same (Tables 1 and 2, respectively), the hypothesized photo-to-thermal pathway in Fig. 4 is validated and the applicability of the TST method is confirmed.

**Table 2:** Thermodynamics of the elementary reactions for the internal base prFMN^im^ → prFMN^ket^ photoisomerization (Type (4) mechanism), obtained from the DFT/B3LYP/DZP, TD-DFT/B3LYP/DZP and TST methods. Δ*G*° and Δ*H*° = Gibbs free energy and enthalpy in kJ mol^−1^; Δ*S*° = entropy in kJ mol^−1^ K^−1^; Δ*G*°^,Tot^ = total reaction Gibbs free energy for prFMN^im^ → prFMN^ket^ and; Δ*S*°^,Rx^ = reaction entropy of all the elementary processes; *T* = temperature in K

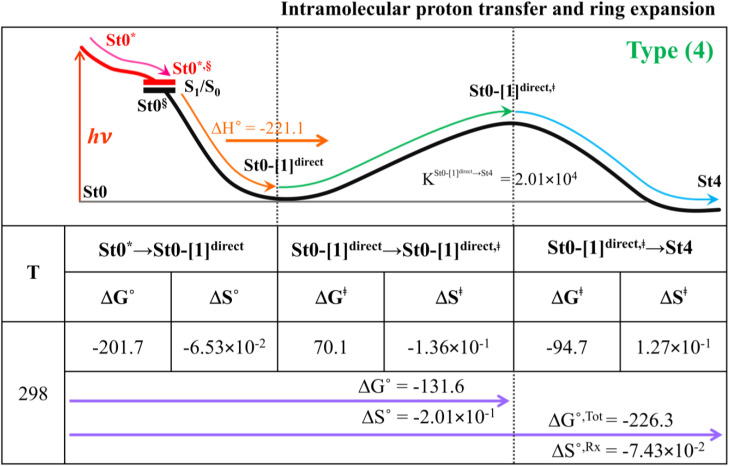

### Kinetics of elementary reactions

To correlate the theoretical results with the experimental data^5^ and to explore the possibility of improving the enzyme efficiency of Fdc1, the rate constants (*k*^Q-vib^) for Type (3) and Type (4) mechanisms (Tables S3 and S4,† respectively) were analyzed and discussed in detail. The experimental rate constants revealed that the enzyme activity of Fdc1 purified in the dark was slightly higher than that under visible light, *k*^cat^ = 9.3 ± 0.1 and 7.6 ± 0.2 s^−1^, respectively, and when Fdc1 was prepared in the dark, the enzyme activity remained constant for many hours. However, direct exposure of the Fdc1 enzyme incubated in the dark with UV radiation at *λ* = 365 nm for ∼5 min led to a complete loss of enzyme activity and a change in the UV-visible spectra.

The experimental results also showed that after purification, Fdc1 incubated on ice resulted in a loss of enzyme stability with a half-life *τ*_1/2_^loss,*T*^ ≈ 30 min. Based on the assumption that the loss of enzyme activity at 273 K follows the first-order kinetics, *k*^loss,*T*^ = 0.693/*τ*_1/2_^loss,*T*^ = 3.85 × 10^−4^ s^−1^. In addition, assuming that the photochemical experiment^5^ was conducted at room temperature, the complete loss of enzyme activity due to UV radiation is approximated to be *τ*^loss,UV^ = 300 s, with the rate constant *k*^loss,UV^ = 1/*τ*^loss,UV^ = 3.33 × 10^−3^ s^−1^ at 298 K. The value of *k*^loss,UV^ is compatible with the rate-determining step in the Type (3) mechanism; for the 
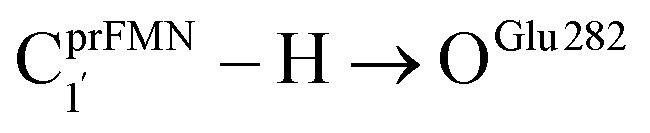
 intermolecular proton transfer from prFMN^im^ to Glu282, *k*_f_^Q-vib^ = 3.53 × 10^−3^ s^−1^ at 298 K (Table S3†).

Although the internal base pathway in Type (4) mechanism is energetically and thermodynamically more favorable than Type (3) mechanism, the concerted rate-determining process is too fast compared with the experimental rate constant, *k*_f_^Q-vib^ = 3.26 × 10^0^ s^−1^ compared with *k*^loss,UV^ = 3.33 × 10^−3^ s^−1^ at 298 K. Therefore, Type (3) mechanism, which requires an external base, is likely to represent the prFMN^im^ → prFMN^ket^ photoisomerization mechanism.

### Balance between the loss of enzyme stability and the efficiency by UV radiation

Based on the above discussion on the theoretical and experimental data,^5^ to reduce the prFMN^im^ → prFMN^ket^ photoisomerization rate without substantial loss of the Fdc1 stability, an appropriate enzymatic decarboxylation temperature should be chosen; an effective enzymatic decarboxylation using Fdc1 could be achieved by slowing down the photo-to-thermal process (formation of the high-energy barrier transition state) in Type (3) mechanism, compared with the enzymatic decarboxylation rate.

**Fig. 8:**
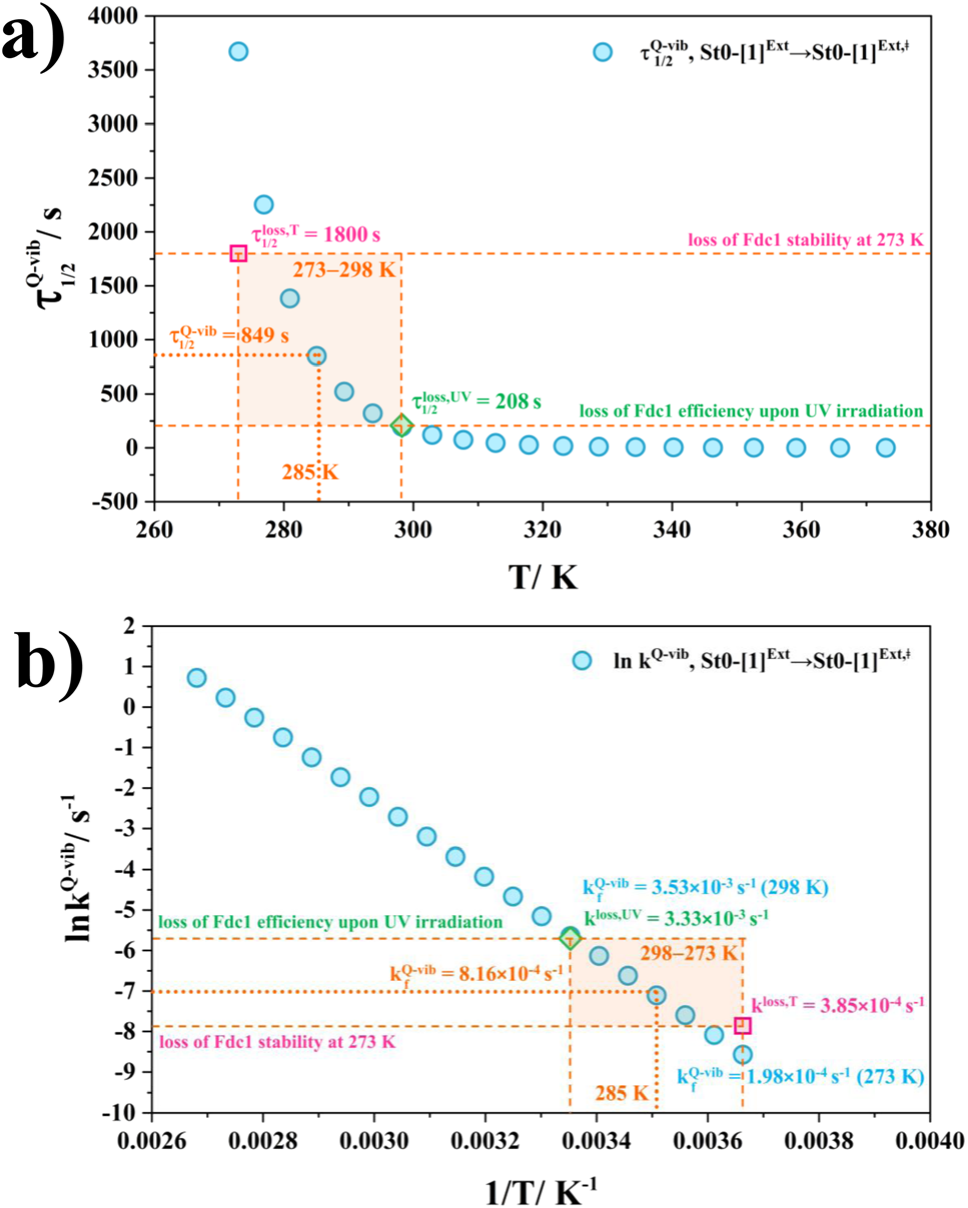
(a) and (b) Correlations between *τ*_1/2_^Q-vib^ and *T*, and ln *k*_f_^Q-vib^ and 1/*T* for the rate determining step (St0-[1]^Ext^ → St0-[1]^Ext,‡^ in Fig. 7(b) in Type (3) mechanism obtained from the TST method, compared with experiment.^5^*τ*_1/2_^loss,*T*^ and *k*^loss,*T*^ = half-life and first-order rate constant for the loss of enzyme stability at 273 K;^5^*τ*_1/2_^loss,UV^ and *k*^loss,UV^ = half-life and first-order rate constant for the loss of enzyme activity due to UV radiation at 365 nm;^5^*τ*_1/2_^Q-vib^ and *k*_f_^Q-vib^ = half-life and first-order rate constant for the rate-determining intermolecular proton transfer (St0-[1]^Ext^ → St0-[1]^Ext,‡^) in Fig. 7(b).

To recommend the appropriate temperature, at which the styrene production is kinetically controlled in the presence of UV radiation, the plot of the half-life for the formation of the high-energy barrier transition state (*τ*_1/2_^Q-vib^) *versus T* and the Arrhenius plot for this photo-to-thermal pathway were constructed over the studied temperature range and are shown in Fig. 8. Fig. 8a shows that *τ*_1/2_^Q-vib^ varies exponentially over the temperature range of 273–298 K. Thus, to balance between the loss of the enzyme stability at 273 K and that under UV irradiation, the appropriate temperature should be in this temperature range, *τ*_1/2_^loss,*T*^ = 1800 (*T* = 273 K) and *τ*_1/2_^loss,UV^ = 208 s (*T* = 298 K).

As a rule of thumb, the rate of chemical reaction doubles if the temperature increases by 10 K. Therefore, the experimental rate constants to produce styrene in this temperature range are *k*^cat^ = 3.00 and 7.60 s^−1^, and the rate constants for the 
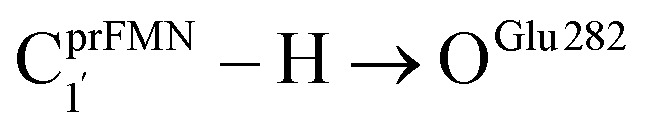
 intermolecular proton transfer (rate determining step for prFMN^im^ → prFMN^ket^) are *k*_f_^Q-vib^ = 1.98 × 10^−4^ s^−1^ (273 K) and 3.53 × 10^−3^ s^−1^ (298 K).

The Arrhenius plot in Fig. 8b reveals that in this temperature range, *k*^loss,*T*^ = 3.85 × 10^−4^ s^−1^ (*T* = 273 K) and *k*^loss,UV^ = 3.33 × 10^−3^ s^−1^ (*T* = 298 K), and the optimal temperature should be at *T* = 285 K with *k*_f_^Q-vib^ = 8.16 × 10^−4^ s^−1^ for the photo-to-thermal pathway. Therefore, the enzyme activity of Fdc1 purified under visible light at 285 K is approximated to be *k*^cat^ ≈ 3.8 s^−1^. This experimental rate constant is ∼4600 times larger than the rate-determining step for prFMN^im^ → prFMN^ket^ at the same temperature, and styrene production becomes kinetically controlled in the presence of UV radiation at this temperature.

## Conclusions

The enzymatic decarboxylation of α,β-unsaturated acids using the Fdc1 enzyme and prFMN cofactor is a potential, environmentally friendly reaction for the biosynthesis of styrene and its derivatives. However, an experiment indicated that the enzyme activity of Fdc1 depends on the ring structure of prFMN, namely, the iminium and ketimine forms, and the loss of the enzyme activity results from the prFMN^im^ → prFMN^ket^ photoisomerization with the light-dependent reaction suggested to occur in the cyclization process.

Herein, to obtain insight into the prFMN^im^ → prFMN^ket^ photoisomerization process and to improve the enzyme efficiency of Fdc1, two proposed photoisomerization mechanisms with different proton sources for the acid–base reaction were studied using theoretical methods. This mechanistic study focused on the photoisomerization pathways in the S_1_ and S_0_ states and on the kinetics and thermodynamics of the photo-to-thermal process in the active site of Fdc1. Analysis of the equilibrium structures of the model active site clusters revealed that because the active site volumes (residue-to-residue distances) obtained from the DFT/B3LYP/DZP geometry optimization were not significantly different, the model active site clusters without the Fdc1 backbone were proved to be reasonable and can be used in the study of the enzymatic decarboxylation process.

The PESs calculated from the DFT/B3LYP/DZP and TD-DFT/B3LYP/DZP methods suggested that the light-dependent reaction occurs in the rate-determining proton transfer process (acid–base reaction), and the mechanism involving intermolecular proton transfer between prFMN^im^ and Glu282 (external base) is energetically more favorable than that involving intramolecular proton transfer in prFMN^im^ (internal base). The light-dependent reaction was confirmed by the calculated UV-visible spectra to be a high-energy-barrier proton transfer process, for which the S_0_ → S_1_ photoexcited precursor is St0 (prFMN^im^ in the active site cluster). The thermodynamic results obtained from the TST method suggested that the exothermic relaxation energy in the photo-to-thermal process in the Type (3) mechanism can promote the spontaneous formation of the high-energy-barrier transition state.

Based on the analysis of the theoretical and experimental data, effective enzymatic decarboxylation of the α,β-unsaturated acids using Fdc1 could be achieved by slowing down the formation of the undesirable thermodynamically favorable product (prFMN^ket^). Because the kinetic results suggested that the rate constant of formation of the high-energy-barrier transition state varies exponentially over the temperature range of 273–298 K, and because the experiment showed that incubating Fdc1 on ice could result in complete loss of enzyme stability, it is recommended to perform the decarboxylation reaction at *T* = 285 K. This temperature allows for kinetically controlled styrene production in the presence of UV radiation, thus addressing the balance between enzyme stability loss at 273 K and under UV radiation. The computational strategy and fundamental insights obtained in this study could serve as guidelines for future theoretical and experimental investigations on the same and similar photochemical systems.

## Conflicts of interest

There are no conflicts to declare.

## Supplementary Material

RA-014-D4RA02035A-s001
