# Supplementary material for: Mechanisms of photoisomerization of the prenylated flavin mononucleotide cofactor: a theoretical study
Source: RSC Adv. 2024 Jun 24;14(28):20061–72. doi: 10.1039/d4ra02035a (PMC11194708; doi:10.1039/d4ra02035a)
Supplement: RA-014-D4RA02035A-s001 [file RA-014-D4RA02035A-s001.pdf]

Electronic Supplementary Material (ESI) for RSC Advances.
This journal is © The Royal Society of Chemistry 2024

Electronic Supplementary Information (ESI)

Mechanisms of photoisomerization of the prenylated flavin mononucleotide cofactor: A theoretical study†

by

Pannipa Panajapo

Phorntep Promma

and

*Kritsana Sagarik**

School of Chemistry

Institute of Science

Suranaree University of Technology

Nakhon Ratchasima 30000

Thailand

*corresponding author: *kritsana@sut.ac.th*

Tel./Fax: (6681) 8783994

Figure S1 The S_0 PESs for the intramolecular proton transfer and concerted covalent bond dissociations (St0→St1^{Int}→St2^{Int}) in Type **(1)** mechanism[5] (Figure 1), obtained from the DFT/B3LYP/DZP reaction path optimizations.

a) $C_{1'}^{prFMN}$–H...$O^{prFMN^{im}}$ intramolecular proton transfer in **prFMN^{im}** (St0→St1^{Int}).

b) Concerted N_5^{prFMN}–C_{5a}^{prFMN} and N_{10}^{prFMN}–C_{9a}^{prFMN} covalent bond dissociations in **prFMN^{im}** (St1^{Int}→St2^{Int}).

a)

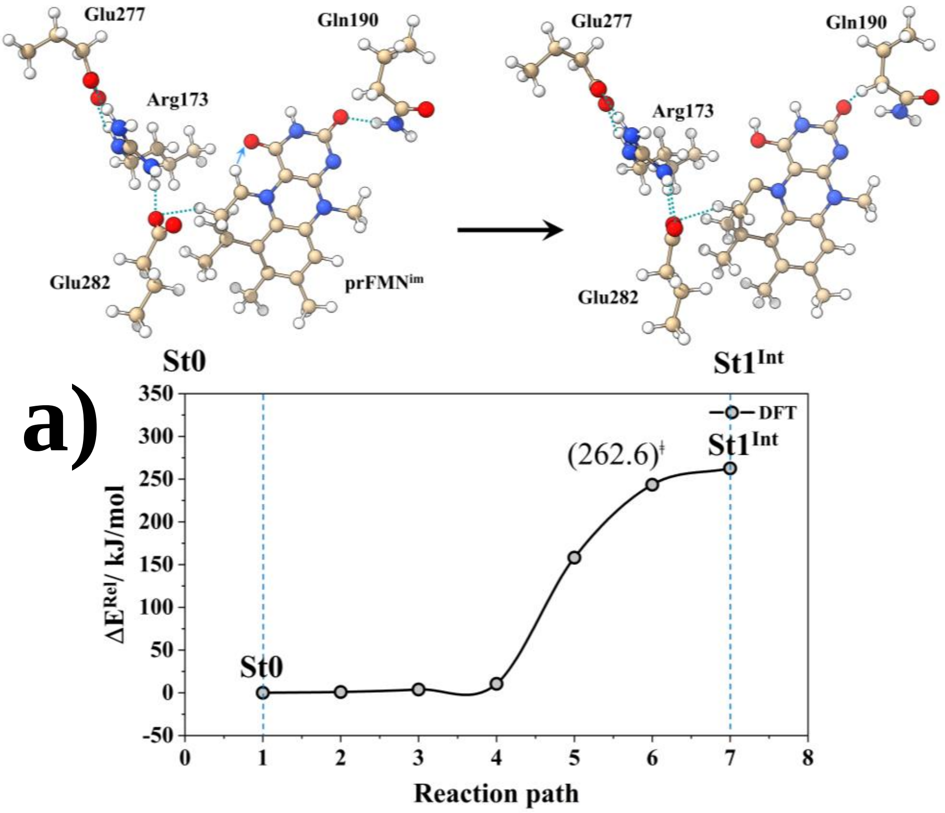
Glu277
Gln190
Arg173
Glu282
prFMNim
St0
St1Int
DFT
(262.6)ǂ
St1Int
St0
ΔERel/ kJ/mol
Reaction path

b)

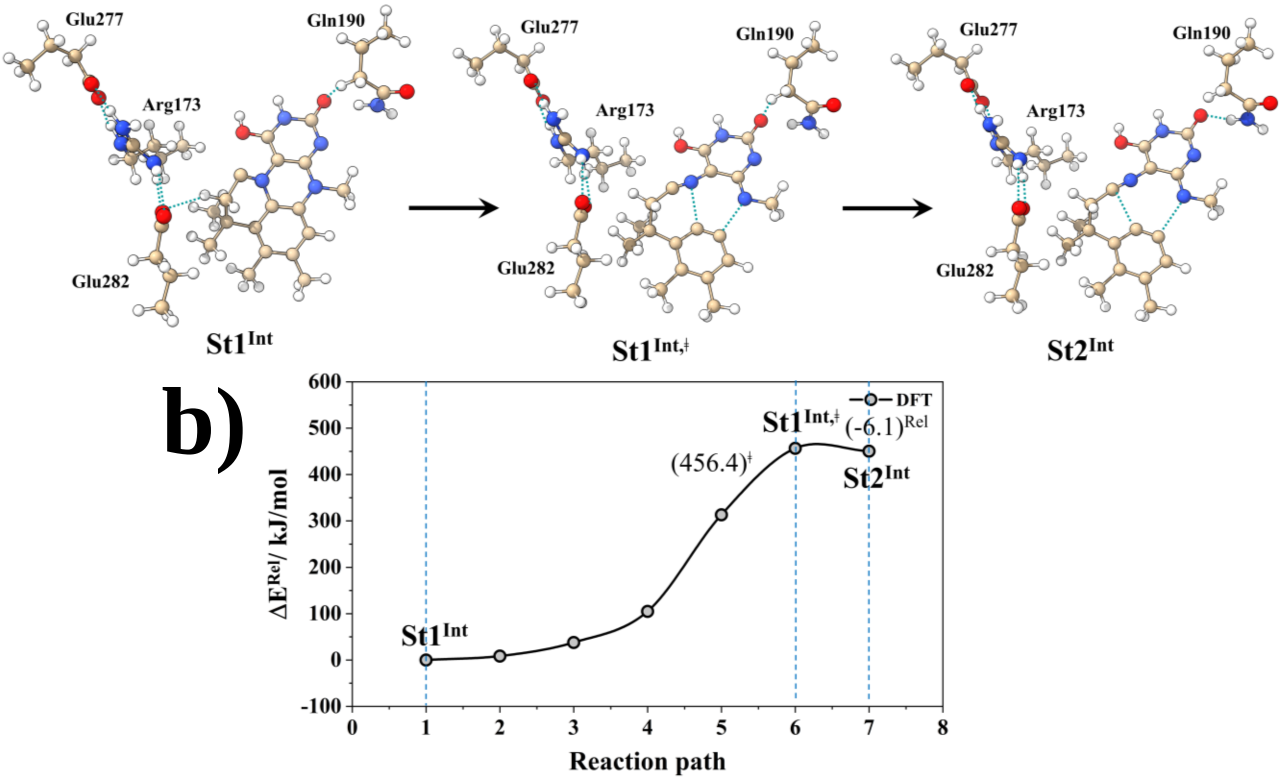
Glu277
Gln190
Arg173
Glu282
St1Int
St1Int,ǂ
St2Int
DFT
St1Int,ǂ
(-6.1)Rel
(456.4)ǂ
St2Int
St1Int
ΔERel/ kJ/mol
Reaction path

Figure S1

Figure S2 The S_0 PESs for the intermolecular proton transfer and concerted covalent bond dissociations (St0→St1^{Ext}→St2^{Ext}) in Type **(2)** mechanism[5] (Figure 1), obtained from the DFT/B3LYP/DZP reaction path optimizations.

a) Intermolecular proton transfer in the $C_{1'}^{prFMN}$–H→O^{Glu282} H-bond between **prFMN^{im}** and **Glu282** (St0→St1^{Ext}).

b) Concerted N_5^{prFMN}–C_{5a}^{prFMN} and N_{10}^{prFMN}–C_{9a}^{prFMN} covalent bond dissociations in **prFMN^{im}** (St1^{Ext}→St2^{Ext}).

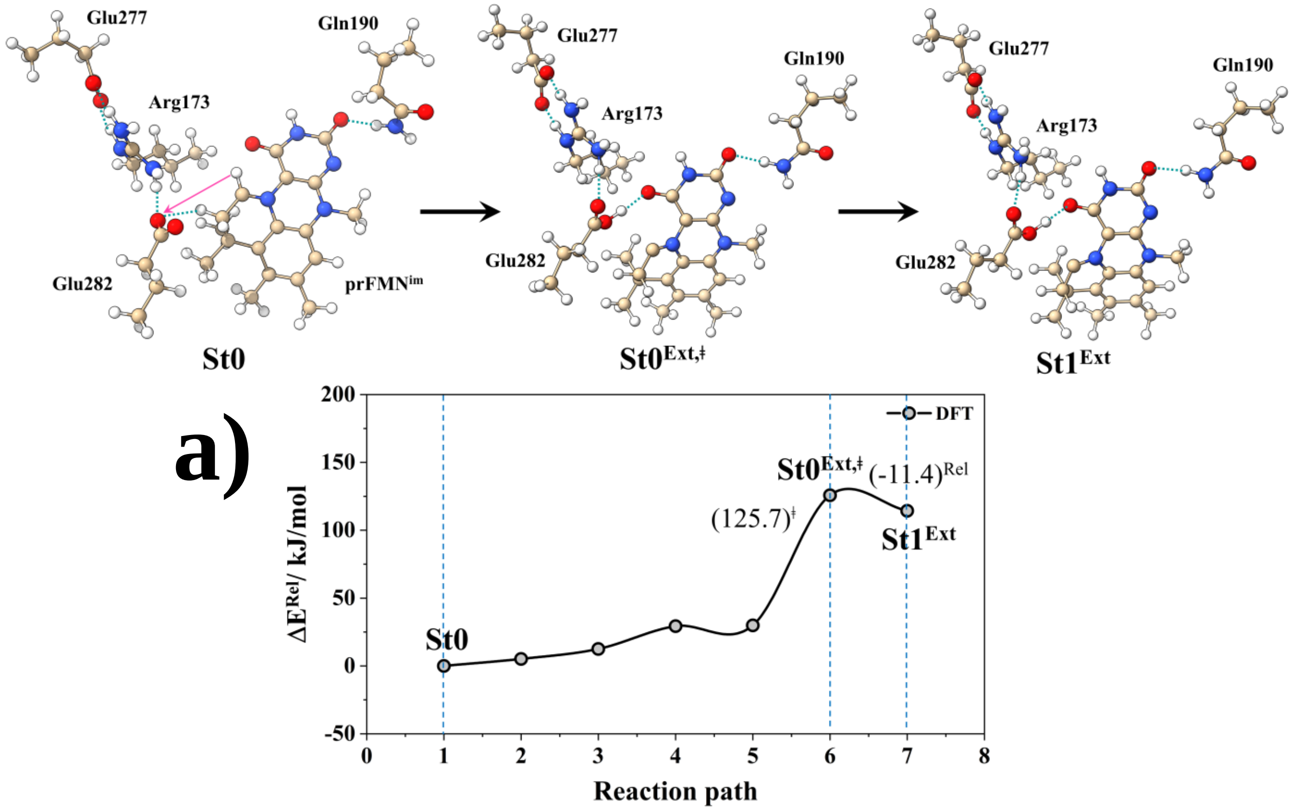
Glu277
Gln190
Arg173
Glu282
prFMNim
St0
St0Ext,‡
St1Ext
a)
DFT
(125.7)‡
(-11.4)Rel
ΔERel/ kJ/mol
Reaction path

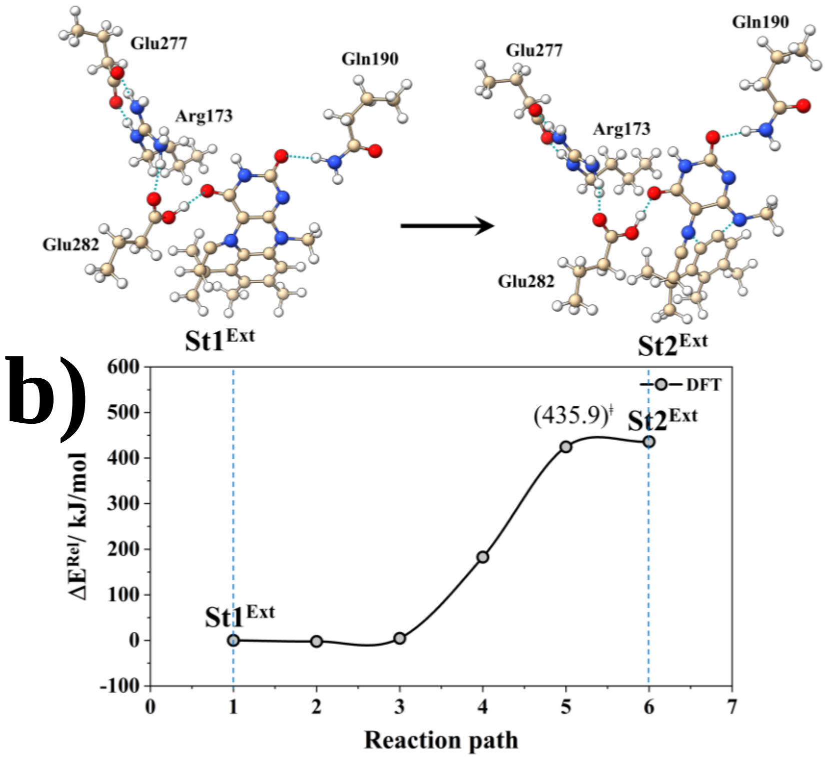
Glu277
Gln190
Arg173
Glu282
St1Ext
St2Ext
b)
DFT
(435.9)‡
ΔERel/ kJ/mol
Reaction path

Figure S2

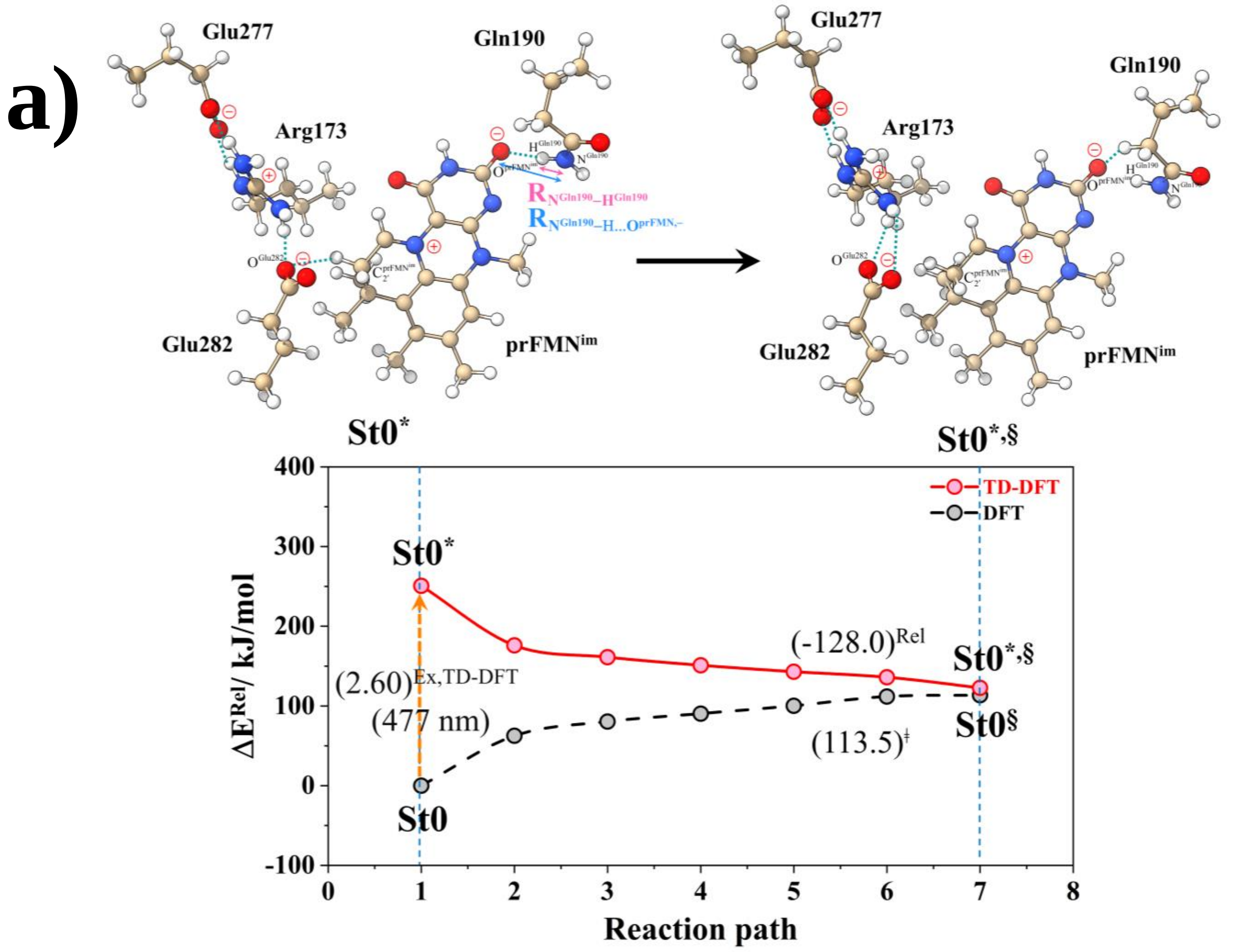

Figure S3 a) The S_1 PES (solid line) for the relaxation of the $S_0 \rightarrow S_1$ vertically excited active site cluster to the structure at the S_0/S_1 intersection ($St0^* \rightarrow St0^{*,\S}$), obtained from TD-DFT/B3LYP/DZP reaction path optimizations; dash line = S_0 PES computed using the geometry on the S_1 PES and DFT/B3LYP/DZP method. b) The variation of the N^{Gln190}–H...$O^{prFMN,-}$ H-bond distances on the S_1 PES, which reflects an increase in the N^{Gln190}–H...$O^{prFMN,-}$ H-bond strength (red-shift) in the S_1 state towards the S_0/S_1 intersection.

b)

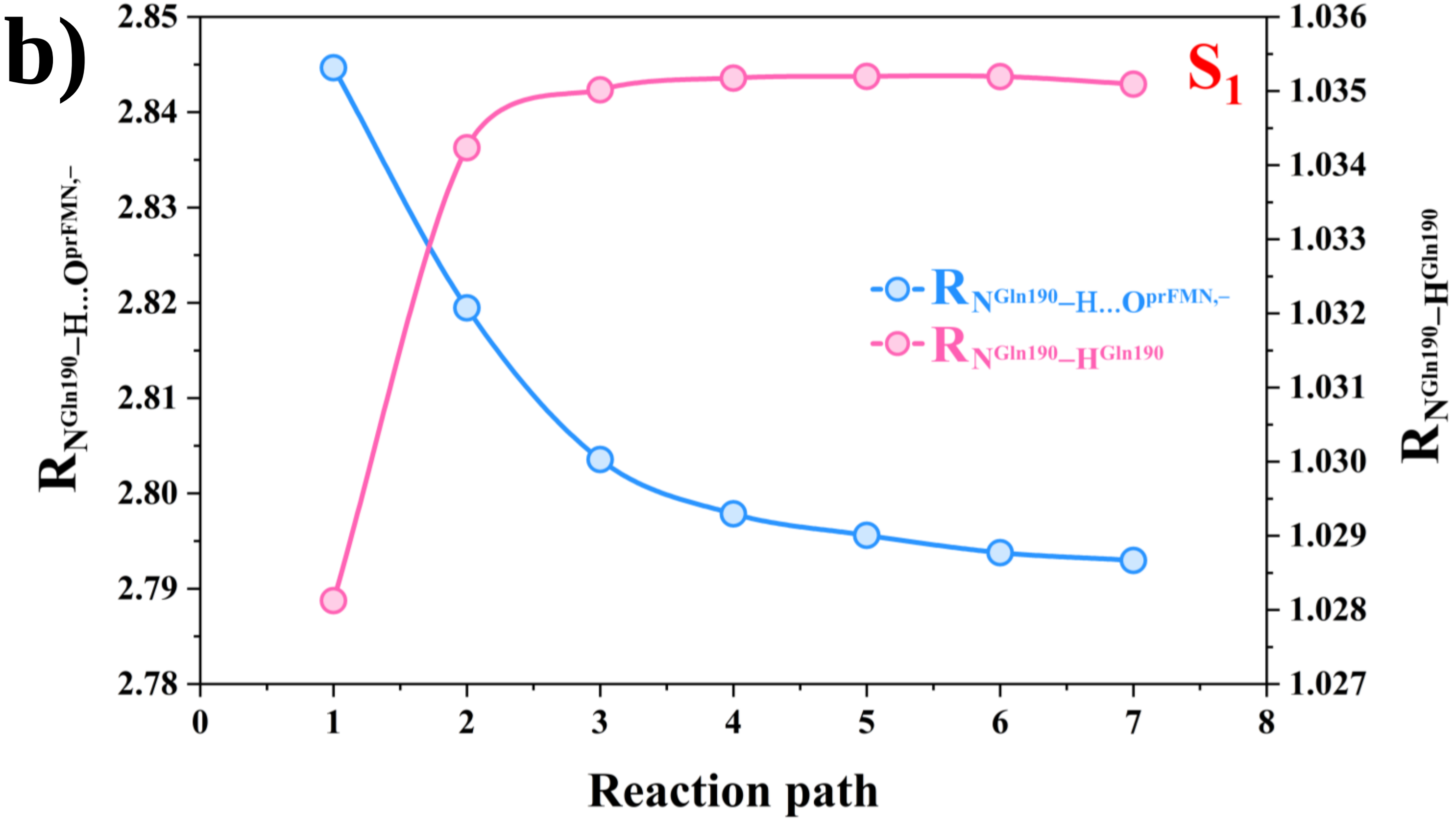

Figure S3 (Cont.)

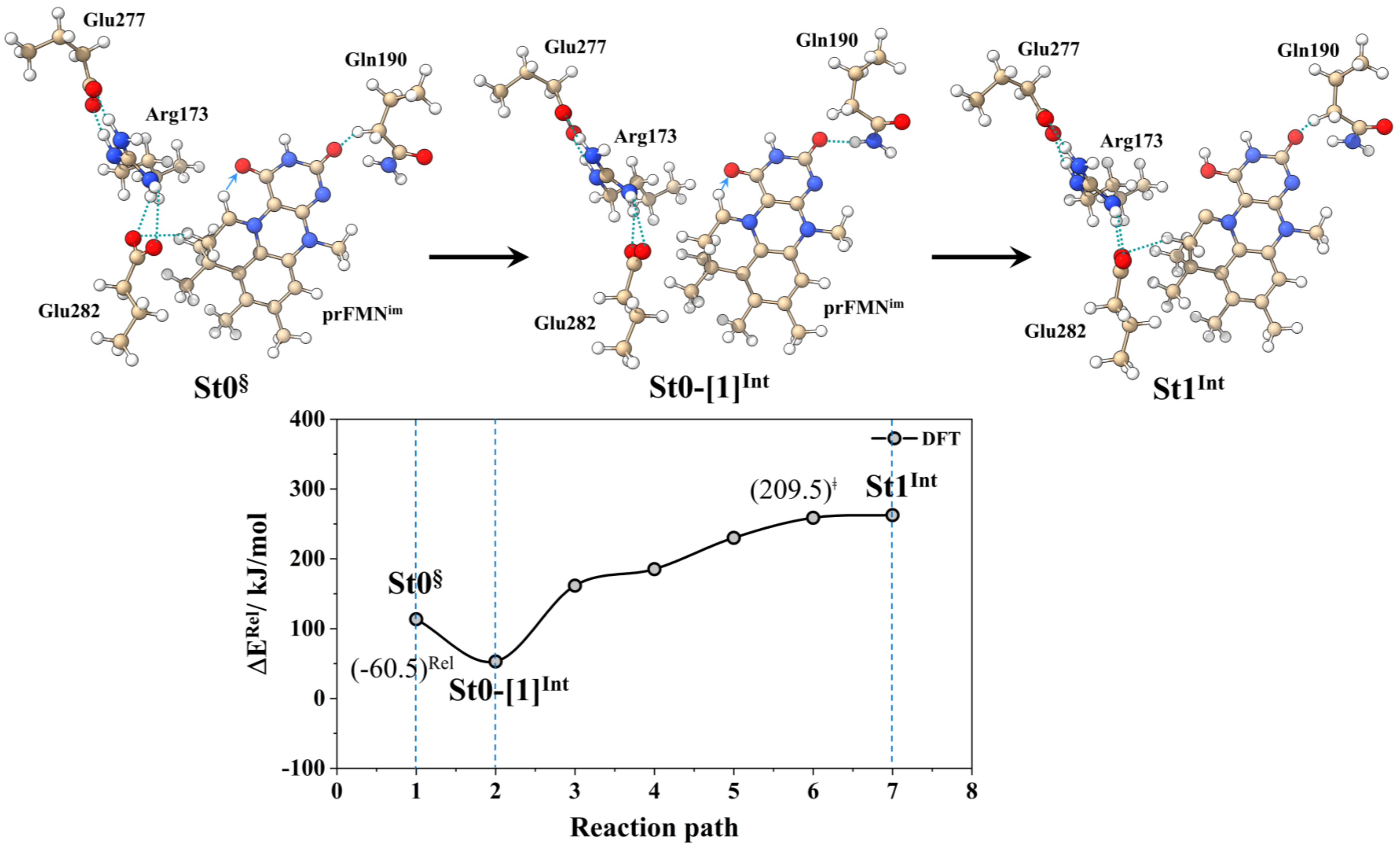

Figure S4 The S_0 PES for the $C_{1'}^{prFMN}$–H→$O^{prFMN^{im}}$ intramolecular proton transfer after the $S_1 \rightarrow S_0$ nonradiative relaxation ($St0^{\S} \rightarrow St1^{Int}$), obtained from DFT/B3LYP/DZP reaction path optimizations.

Figure S5 The S_0 PESs for formation of **prFMN$^{\mathbf{ket}}$** after the $S_1 \rightarrow S_0$ nonradiative relaxation in Type **(3)** mechanism (St0$^{\S}\rightarrow$ St4), obtained from DFT/B3LYP/DZP reaction path optimizations.

a) $C_{1'}^{prFMN}$–H→O^{Glu282} intermolecular proton transfer (St0$^{\S}\rightarrow$St1^{Ext}).

b) Ring expansion (St1$^{Ext}\rightarrow$St3^{Ext})

c) Reverse protonation (St3$^{Ext}\rightarrow$St4).

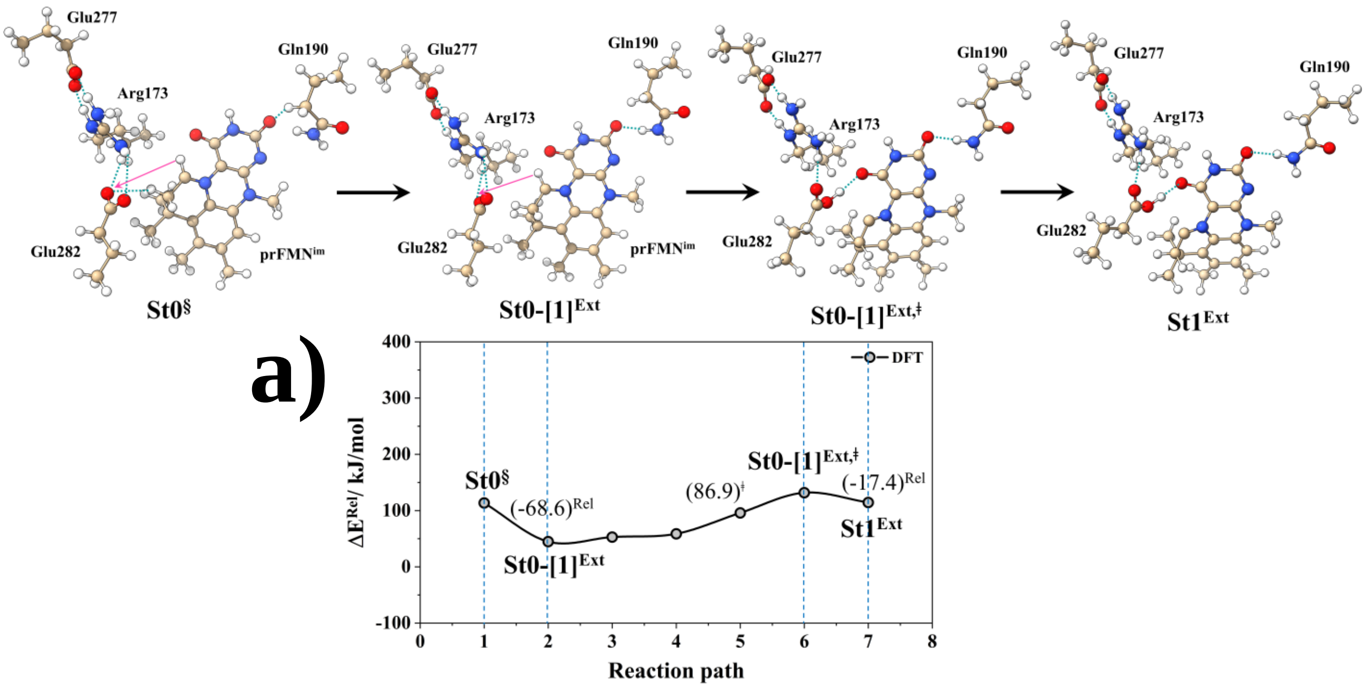

a)
Glu277
Arg173
Gln190
Glu282
prFMNim
St0§
St0-[1]Ext
St0-[1]Ext,‡
St1Ext
DFT
ΔERel/ kJ/mol
Reaction path
(-68.6)Rel
(86.9)‡
(-17.4)Rel

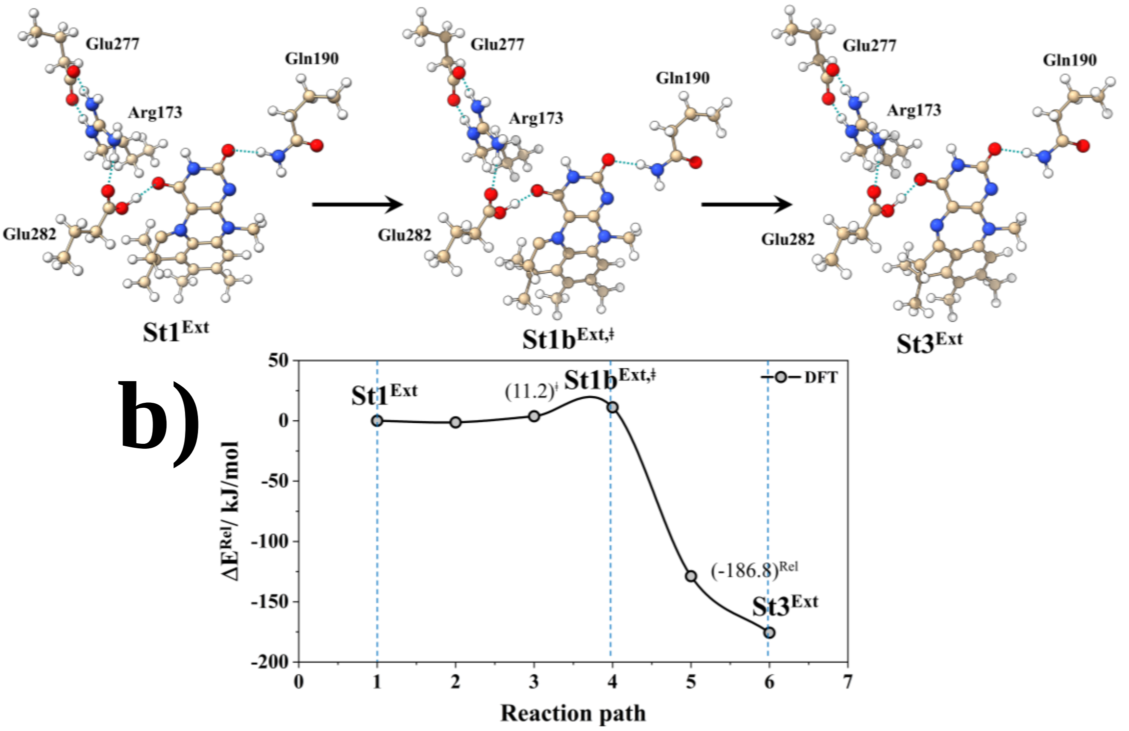

b)
Glu277
Arg173
Gln190
Glu282
St1Ext
St1bExt,‡
St3Ext
DFT
ΔERel/ kJ/mol
Reaction path
(11.2)‡
(-186.8)Rel

Figure S5

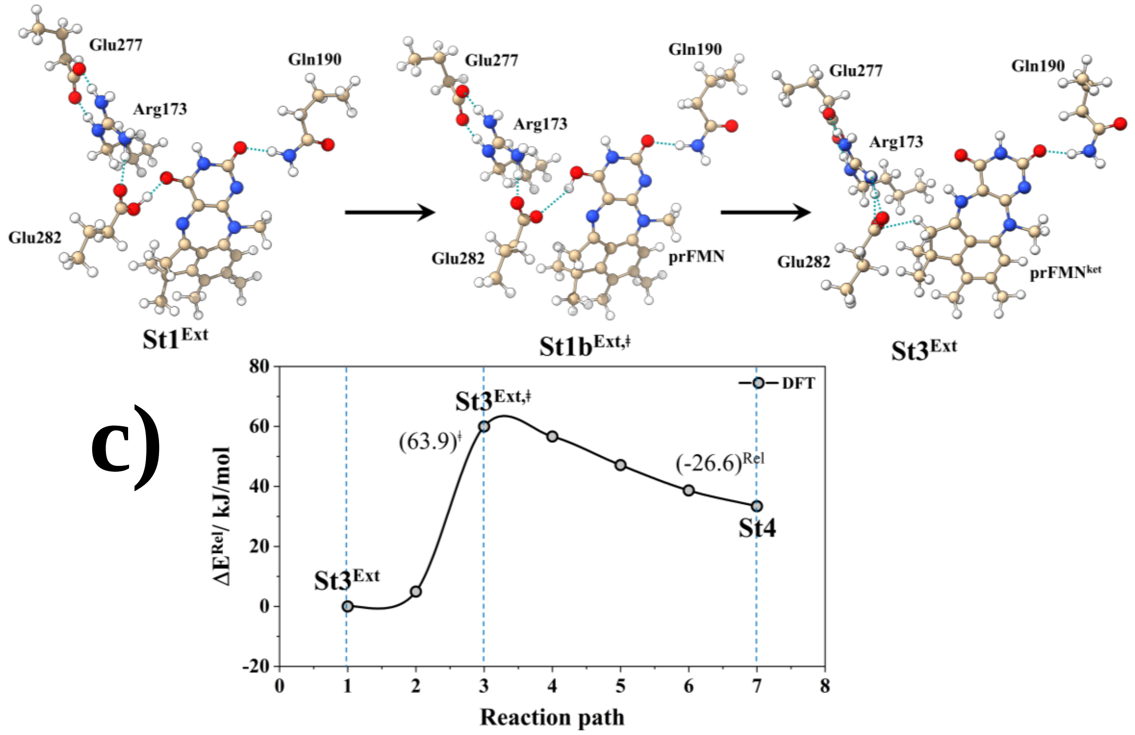
Glu277
Gln190
Arg173
Glu282
St1Ext
St1bExt,‡
prFMN
St3Ext
prFMNket
c)
DFT
St3Ext,‡
(63.9)‡
(-26.6)Rel
St3Ext
St4
ΔERel/ kJ/mol
Reaction path
80
60
40
20
0
-20
0
1
2
3
4
5
6
7
8

Figure S5

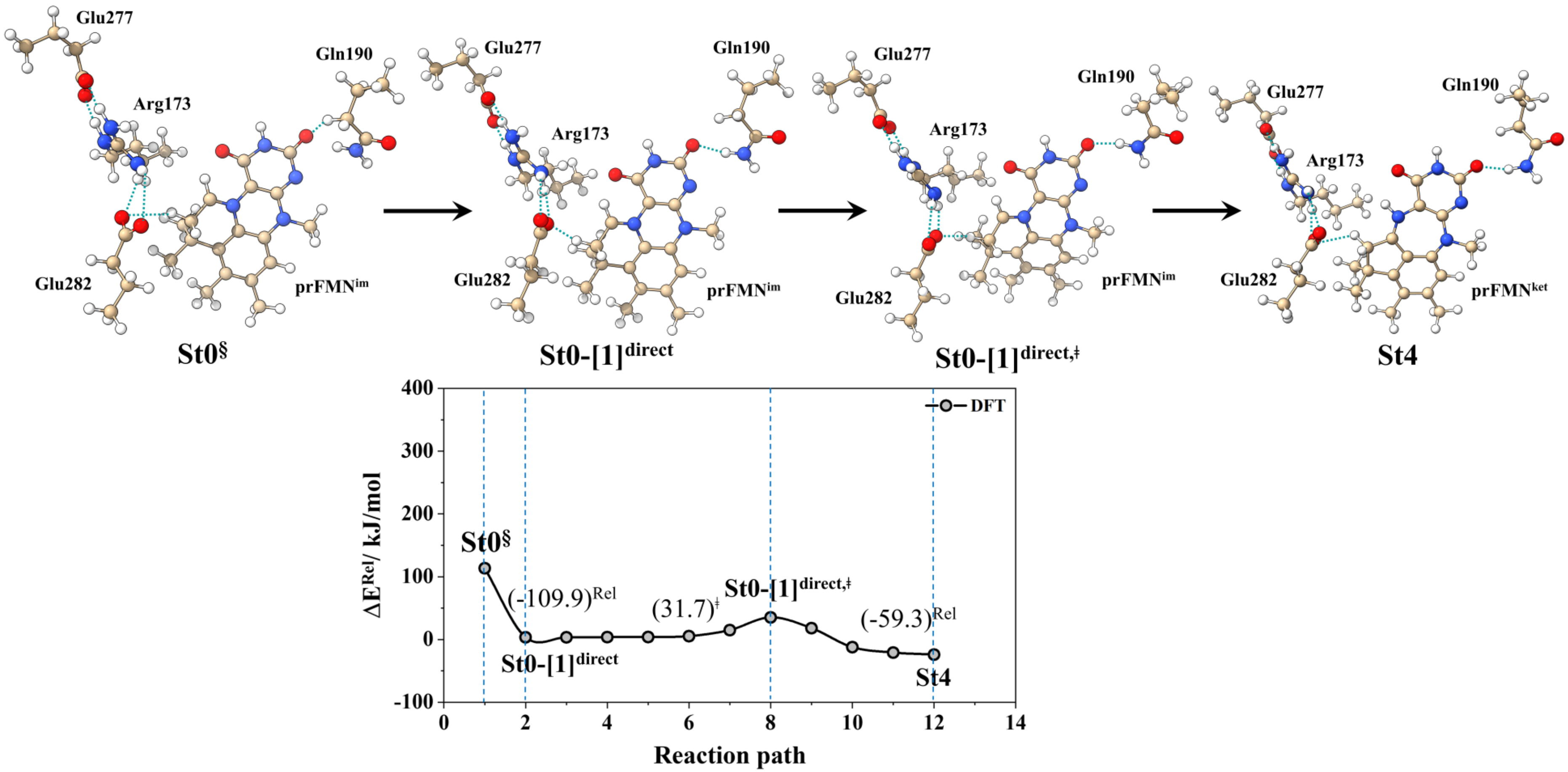

Figure S6 The S_0 PES for formation of **prFMN^{ket}** after the $S_1 \rightarrow S_0$ nonradiative relaxation in Type **(4)** mechanism (St0$^{\S} \rightarrow$ St4). The $S_1 \rightarrow S_0$ nonradiative relaxation is followed by concerted $C_{1'}^{prFMN}$–H→N_5^{prFMN} intramolecular proton transfer and ring expansion (St0$^{\S} \rightarrow$St4), obtained from DFT/B3LYP/DZP reaction path optimization.

Table S1 Equilibrium structures and energies of molecules involved in the **prFMN^{im}→prFMN^{ket}** photoisomerization obtained from DFT/B3LYP/DZP geometry optimizations. E^{Tot} = total energy in au; ΔE^{Ex} = the $S_0 \rightarrow S_1$ energy in eV; (…) = absorption wavelength in nm.

**Structures**	**E^{Tot}/ au**	**ΔE^{Ex}/ eV**
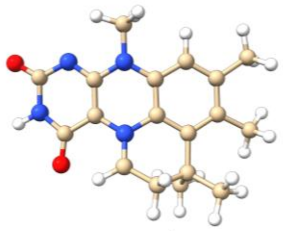 **prFMN^{im}**	-1066.743681	3.15 (394 nm)
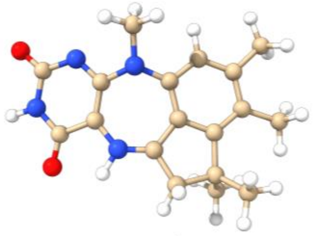 **prFMN^{ket}**	-1066.756385	2.25 (551 nm)
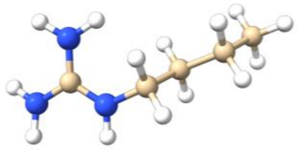 **Arg173**	-362.765659	7.17 (173 nm)
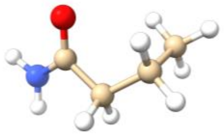 **Gln190**	-287.647485	5.75 (215 nm)
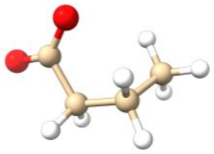 **Glu277**	-306.937343	5.23 (237 nm)
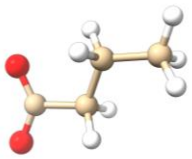 **Glu282**	-306.935420	4.79 (259 nm)

Table S2 a) Equilibrium structures, energies and residue-to-residue (R-to-R) distances in the active site clusters on the **prFMN^{im}→prFMN^{ket}** photoisomerization pathways obtained from DFT/B3LYP/DZP geometry optimizations.

b) The average residue-to-residue distances and standard deviation (SD) for all the equilibrium structures of the active site clusters.

E^{Tot} = total energy in au; ΔE^{Ex} = the $S_0 \rightarrow S_1$ energy in eV; (…) = absorption wavelength in nm; sp = single point calculation.

Table S2a

Model molecular cluster	E^{Tot}/ au	ΔE^{Ex}/ eV	R-to-R/ Å
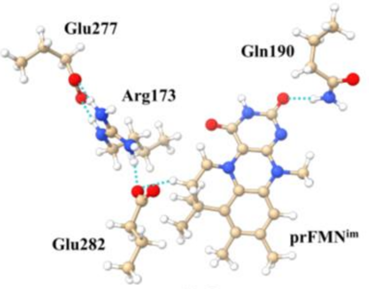 **St0**	-2331.560638	2.60 (477 nm)	$R_{C_R^{Gln190}-C_R^{Arg173}} = 12.40$<br>$R_{C_R^{Gln190}-C_R^{Glu277}} = 17.47$<br>$R_{C_R^{Gln190}-C_R^{Glu282}} = 17.13$<br>$R_{C_R^{Arg173}-C_R^{Glu277}} = 9.79$<br>$R_{C_R^{Arg173}-C_R^{Glu282}} = 10.11$<br>$R_{C_R^{Glu277}-C_R^{Glu282}} = 13.25$
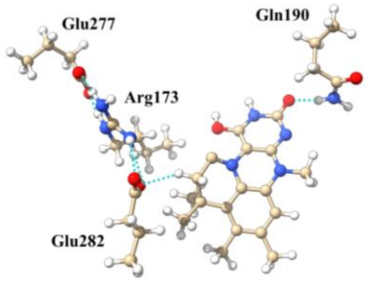 **St1^{Int}**	-2331.460634	2.69 (462 nm)	$R_{C_R^{Gln190}-C_R^{Arg173}} = 12.42$<br>$R_{C_R^{Gln190}-C_R^{Glu277}} = 17.48$<br>$R_{C_R^{Gln190}-C_R^{Glu282}} = 17.14$<br>$R_{C_R^{Arg173}-C_R^{Glu277}} = 9.82$<br>$R_{C_R^{Arg173}-C_R^{Glu282}} = 10.10$<br>$R_{C_R^{Glu277}-C_R^{Glu282}} = 13.26$
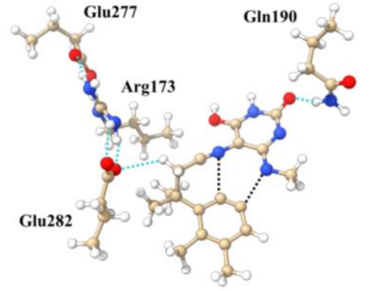 **St2^{Int}**	-2331.289127^{sp}	2.96 (419 nm)	$R_{C_R^{Gln190}-C_R^{Arg173}} = 12.40$<br>$R_{C_R^{Gln190}-C_R^{Glu277}} = 17.47$<br>$R_{C_R^{Gln190}-C_R^{Glu282}} = 17.14$<br>$R_{C_R^{Arg173}-C_R^{Glu277}} = 9.80$<br>$R_{C_R^{Arg173}-C_R^{Glu282}} = 10.11$<br>$R_{C_R^{Glu277}-C_R^{Glu282}} = 13.74$
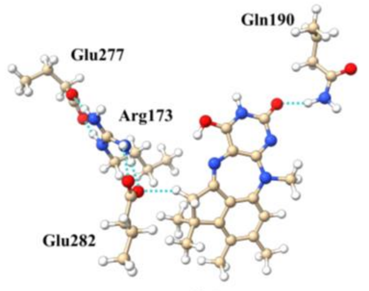 **St3^{Int}**	-2331.559782	2.70 (460 nm)	$R_{C_R^{Gln190}-C_R^{Arg173}} = 12.58$<br>$R_{C_R^{Gln190}-C_R^{Glu277}} = 17.60$<br>$R_{C_R^{Gln190}-C_R^{Glu282}} = 17.20$<br>$R_{C_R^{Arg173}-C_R^{Glu277}} = 10.00$<br>$R_{C_R^{Arg173}-C_R^{Glu282}} = 10.05$<br>$R_{C_R^{Glu277}-C_R^{Glu282}} = 13.34$
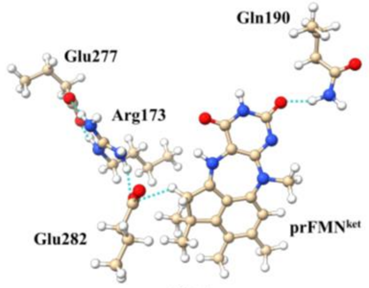 **St4**	-2331.569793	2.27 (546 nm)	$R_{C_R^{Gln190}-C_R^{Arg173}} = 12.48$<br>$R_{C_R^{Gln190}-C_R^{Glu277}} = 17.52$<br>$R_{C_R^{Gln190}-C_R^{Glu282}} = 17.11$<br>$R_{C_R^{Arg173}-C_R^{Glu277}} = 9.98$<br>$R_{C_R^{Arg173}-C_R^{Glu282}} = 10.04$<br>$R_{C_R^{Glu277}-C_R^{Glu282}} = 13.35$

Table S2a (Cont.)

Model molecular cluster	E^{Tot}/ au	ΔE^{Ex}/ eV	R-to-R/ Å
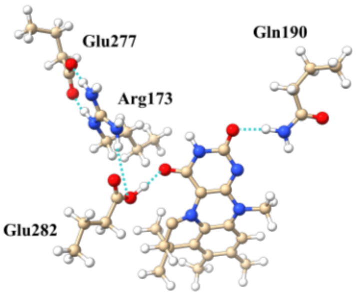 **St1^{Ext}**	-2331.560638	3.21<br>(386 nm)	$R_{C_R^{Gln190}-C_R^{Arg173}} = 12.48$<br>$R_{C_R^{Gln190}-C_R^{Glu277}} = 17.52$<br>$R_{C_R^{Gln190}-C_R^{Glu282}} = 17.11$<br>$R_{C_R^{Arg173}-C_R^{Glu277}} = 9.98$<br>$R_{C_R^{Arg173}-C_R^{Glu282}} = 10.04$<br>$R_{C_R^{Glu277}-C_R^{Glu282}} = 13.35$
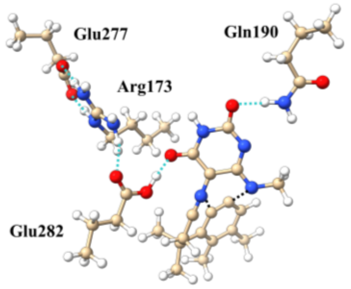 **St2^{Ext}**	-2331.351063^{sp}	1.88<br>(658 nm)	$R_{C_R^{Gln190}-C_R^{Arg173}} = 12.39$<br>$R_{C_R^{Gln190}-C_R^{Glu277}} = 17.47$<br>$R_{C_R^{Gln190}-C_R^{Glu282}} = 17.11$<br>$R_{C_R^{Arg173}-C_R^{Glu277}} = 9.79$<br>$R_{C_R^{Arg173}-C_R^{Glu282}} = 10.08$<br>$R_{C_R^{Glu277}-C_R^{Glu282}} = 13.22$
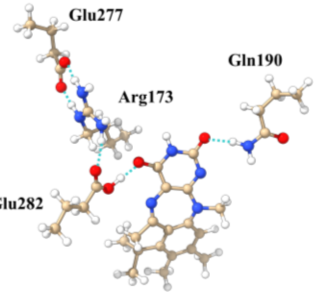 **St3^{Ext}**	-2331.582516	2.67<br>(465 nm)	$R_{C_R^{Gln190}-C_R^{Arg173}} = 12.41$<br>$R_{C_R^{Gln190}-C_R^{Glu277}} = 17.43$<br>$R_{C_R^{Gln190}-C_R^{Glu282}} = 17.02$<br>$R_{C_R^{Arg173}-C_R^{Glu277}} = 9.93$<br>$R_{C_R^{Arg173}-C_R^{Glu282}} = 9.96$<br>$R_{C_R^{Glu277}-C_R^{Glu282}} = 13.21$

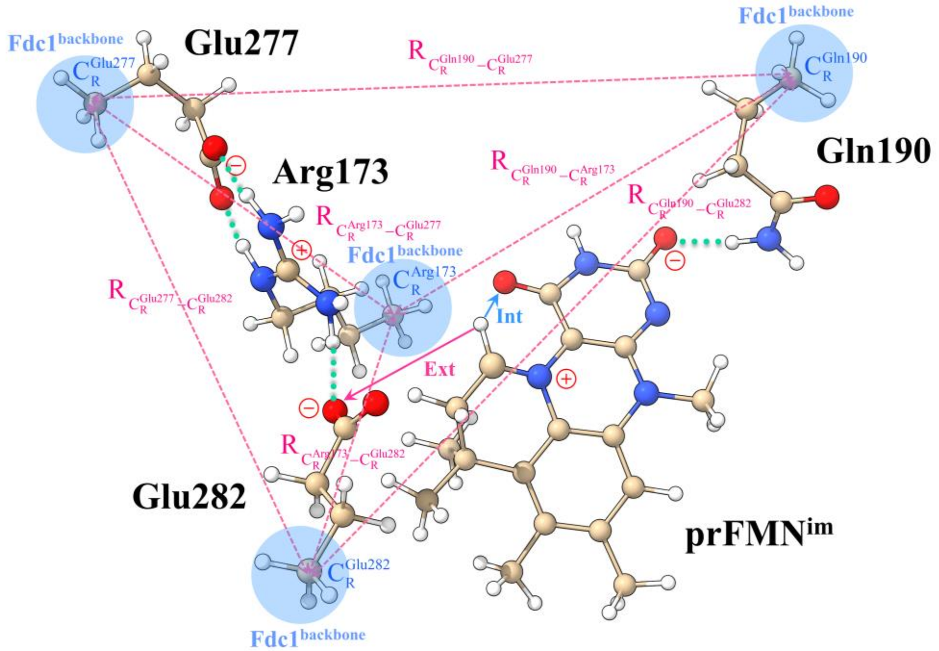

Table S2b

Model molecular cluster	$R_{C_R^{Gln190}-C_R^{Arg173}}$	$R_{C_R^{Gln190}-C_R^{Glu277}}$	$R_{C_R^{Gln190}-C_R^{Glu282}}$	$R_{C_R^{Arg173}-C_R^{Glu277}}$	$R_{C_R^{Arg173}-C_R^{Glu282}}$	$R_{C_R^{Glu277}-C_R^{Glu282}}$
**St0**	12.40	17.47	17.13	9.79	10.11	13.25
**St1^{Int}**	12.42	17.48	17.14	9.82	10.10	13.26
**St3^{Int}**	12.58	17.60	17.20	10.00	10.05	13.34
**St4**	12.48	17.52	17.11	9.98	10.04	13.35
**St1^{Ext}**	12.80	17.51	17.06	9.92	9.91	13.12
**St3^{Ext}**	12.41	17.43	17.02	9.93	9.96	13.21
**Average±SD**	12.52±0.15	17.50±0.06	17.11±0.06	9.91±0.08	10.03±0.08	13.26±0.09

Table S3 Thermodynamics and kinetics of the elementary reactions for the **prFMN^{im}→prFMN^{ket}** photoisomerization (Type **(3)** mechanism), obtained from DFT/B3LYP/DZP reaction path optimizations and TST methods. Rate constants, temperatures and energies are in s^{-1}, K and kJ/mol, respectively; $\Delta E^{\ddagger}$ = energy barriers; $\Delta E^{\ddagger,ZPC}$ = energy barrier with the zero-point vibrational energy; $\Delta H^{\ddagger}$ = activation enthalpy; T_c = crossover temperature; T = temperature; $k_{f/r}^{Q\text{-}Vib}$ = rate constant obtained with quantized vibration correction; $\Delta G^{\ddagger}$ = activation free energy; $\Delta S^{\ddagger}$ = activation entropy; f/r = forward or reverse direction.

Table S3

$S_1 \rightarrow S_0$ relaxation	$\Delta E^{‡}$	$\Delta E^{‡,ZPC}$	$\Delta H^{‡}$	T	$k_{f/r}^{Q\text{-}Vib}$	$\Delta G^{‡}$	$\Delta S^{‡}$
				273	1.61×10^{-7}	102.2	3.46×10^{-2}
				277	3.22×10^{-7}	102.1	3.44×10^{-2}
**St0* ← St0*,§**	119.7	107.6	111.6	298	1.03×10^{-5}	101.5	3.37×10^{-2}
				303	2.05×10^{-5}	101.4	3.36×10^{-2}
				353	1.06×10^{-2}	100.2	3.24×10^{-2}
**Intermolecular proton transfer**	$\Delta E^{‡}$	$\Delta E^{‡,ZPC}$	$\Delta H^{‡}$	T	$k_{f/r}^{Q\text{-}Vib}$	$\Delta G^{‡}$	$\Delta S^{‡}$
				273	4.33×10^{0}	63.3	1.26×10^{-2}
				277	6.56×10^{0}	63.3	1.25×10^{-2}
**St0§ ← St0-[1]^{Ext}**	68.6	66.1	66.8	298	5.22×10^{1}	63.2	1.18×10^{-2}
				303	7.91×10^{1}	63.2	1.17×10^{-2}
				353	3.32×10^{3}	63.1	1.05×10^{-2}
				273	1.89×10^{-4}	86.1	-2.74×10^{-2}
				277	3.08×10^{-4}	86.3	-2.75×10^{-2}
**St0-[1]^{Ext} → St0-[1]^{Ext,‡}**	86.9	78.7	78.7	298	3.53×10^{-3}	87.0	-2.82×10^{-2}
				303	5.76×10^{-3}	87.2	-2.83×10^{-2}
				353	4.71×10^{-1}	89.1	-2.95×10^{-2}
				273	2.17×10^{16}	-18.7	1.28×10^{-1}
				277	2.44×10^{16}	-19.2	1.28×10^{-1}
**St0-[1]^{Ext,‡} ← St1^{Ext}**	17.4	17.4	16.3	298	4.39×10^{16}	-22.0	1.28×10^{-1}
				303	4.93×10^{16}	-22.6	1.28×10^{-1}
				353	1.60×10^{17}	-29.8	1.28×10^{-1}

Table S3 (Cont.)

**Ring expansion**	$\Delta E^{\ddagger}$	$\Delta E^{\ddagger,ZPC}$	$\Delta H^{\ddagger}$	T	$k_{f/r}^{Q\text{-}Vib}$	$\Delta G^{\ddagger}$	$\Delta S^{\ddagger}$
				273	4.87×10^{10}	10.8	1.53×10^{-3}
				277	5.22×10^{10}	10.8	1.41×10^{-3}
**$St1^{Ext} \rightarrow St1^{Ext,\ddagger}$**	11.2	10.3	11.2	298	7.40×10^{10}	11.0	7.85×10^{-4}
				303	7.94×10^{10}	11.0	6.56×10^{-4}
				353	1.49×10^{11}	11.4	-6.04×10^{-4}
				273	1.84×10^{-25}	195.9	-3.66×10^{-2}
				277	5.84×10^{-25}	196.1	-3.67×10^{-2}
**$St1^{Ext,\ddagger} \leftarrow St3^{Ext}$**	186.8	186.2	186.0	298	1.89×10^{-22}	197.1	-3.73×10^{-2}
				303	6.00×10^{-22}	197.3	-3.74×10^{-2}
				353	1.98×10^{-17}	199.6	-3.87×10^{-2}
**Reverse protonation**	$\Delta E^{\ddagger}$	$\Delta E^{\ddagger,ZPC}$	$\Delta H^{\ddagger}$	T	$k_{f/r}^{Q\text{-}Vib}$	$\Delta G^{\ddagger}$	$\Delta S^{\ddagger}$
				273	6.10×10^{7}	26.0	1.31×10^{-1}
				277	9.11×10^{7}	25.5	1.31×10^{-1}
**$St3^{Ext} \rightarrow St3^{Ext,\ddagger}$**	63.9	62.8	61.8	298	6.75×10^{8}	22.6	1.31×10^{-1}
				303	1.01×10^{9}	22.0	1.31×10^{-1}
				353	3.73×10^{10}	15.5	1.31×10^{-1}
				273	1.75×10^{11}	7.9	4.27×10^{-2}
				277	1.97×10^{11}	7.8	4.26×10^{-2}
**$St3^{Ext,\ddagger} \leftarrow St4$**	26.6	17.2	19.6	298	3.61×10^{11}	7.1	4.19×10^{-2}
				303	4.07×10^{11}	6.9	4.18×10^{-2}
				353	1.22×10^{12}	5.3	4.05×10^{-2}

Table S4 Thermodynamics and kinetics of the elementary reactions for the **prFMN^{im}→prFMN^{ket}** photoisomerization (Type **(4)** mechanism), obtained from DFT/B3LYP/DZP reaction path optimizations and TST methods. Rate constants, temperatures and energies are in s^{-1}, K and kJ/mol, respectively; $\Delta E^{\ddagger}$ = energy barriers; $\Delta E^{\ddagger,ZPC}$ = energy barrier with the zero-point vibrational energy; $\Delta H^{\ddagger}$ = activation enthalpy; T_c = crossover temperature; T = temperature; $k_{f/r}^{Q\text{-}Vib}$ = rate constant obtained with quantized vibration correction; $\Delta G^{\ddagger}$ = activation free energy; $\Delta S^{\ddagger}$ = activation entropy; f/r = forward or reverse direction.

Table S4

Intramolecular proton transfer and ring expansion	$\Delta E^{\ddagger}$	$\Delta E^{\ddagger,ZPC}$	$\Delta H^{\ddagger}$	T	$k_{f/r}^{Q\text{-}Vib}$	$\Delta G^{\ddagger}$	$\Delta S^{\ddagger}$
				273	3.05×10^{-7}	100.7	3.23×10^{-2}
				277	6.01×10^{-7}	100.6	3.22×10^{-2}
**St0§ ← St0-[1]^{direct}**	109.9	107.0	109.5	298	1.81×10^{-5}	100.1	3.16×10^{-2}
				303	3.57×10^{-5}	100.0	3.14×10^{-2}
				353	1.64×10^{-2}	98.9	3.02×10^{-2}
				273	1.08×10^{0}	66.5	-1.35×10^{-1}
				277	1.30×10^{0}	67.0	-1.35×10^{-1}
**St0-[1]^{direct} → St0-[1]^{direct,‡}**	31.7	29.0	29.7	298	3.26×10^{0}	70.1	-1.36×10^{-1}
				303	3.92×10^{0}	70.8	-1.36×10^{-1}
				353	2.07×10^{1}	78.0	-1.37×10^{-1}
				273	1.91×10^{-5}	91.3	-1.26×10^{-1}
				277	2.73×10^{-5}	91.8	-1.26×10^{-1}
**St0-[1]^{direct,‡} ← St4**	59.3	57.2	56.9	298	1.60×10^{-4}	94.7	-1.27×10^{-1}
				303	2.28×10^{-4}	95.4	-1.27×10^{-1}
				353	5.51×10^{-3}	102.1	-1.28×10^{-1}

Table S5 Thermodynamic properties of the elementary reactions in Type **(3)** and Type **(4)** mechanisms, obtained based on the hypothesized photo-to-thermal pathway in Figure 4 and from the DFT/B3LYP/DZP, TD-DFT/B3LYP/DZP and TST methods.

**a) Type (3)**	**St0*→St0-[1]^{Ext} (I)*→(III)**		**St0*→St0-[1]^{Ext,‡} (I)*→(IV)‡**		**St0*→St1^{Ext} (I)*→(V)**		**St0*→St3^{Ext}**		**St0*→St4**	
**T**	**ΔG°**	**ΔS°**	**ΔG°**	**ΔS°**	**ΔG°**	**ΔS°**	**ΔG°**	**ΔS°**	**ΔG$^{\circ,Tot}$**	**ΔS$^{\circ,Rx}$**
273	-165.5	-4.72×10^{-2}	-79.4	-7.45×10^{-2}	-60.66	-2.03×10^{-1}	-245.8	-1.65×10^{-1}	-227.7	-7.61×10^{-2}
277	-165.4	-4.69×10^{-2}	-79.1	-7.44×10^{-2}	-59.90	-2.03×10^{-1}	-245.2	-1.65×10^{-1}	-227.5	-7.59×10^{-2}
298	-164.8	-4.56×10^{-2}	-77.7	-7.37×10^{-2}	-55.75	-2.02×10^{-1}	-241.9	-1.64×10^{-1}	-226.3	-7.45×10^{-2}
303	-164.6	-4.53×10^{-2}	-77.4	-7.36×10^{-2}	-54.85	-2.02×10^{-1}	-241.1	-1.64×10^{-1}	-226.0	-7.43×10^{-2}
353	-163.3	-4.28×10^{-2}	-74.2	-7.24×10^{-2}	-45.26	-2.01×10^{-1}	-233.4	-1.63×10^{-1}	-223.2	-7.18×10^{-2}

**b) Type (4)**	**St0*→St0-[1]^{Ext} (I)*→(III)**		**St0*→St0-[1]^{Ext,‡} (I)*→(IV)‡**		**St0*→St1 (I)*→(V)**	
**T**	**ΔG°**	**ΔS°**	**ΔG°**	**ΔS°**	**ΔG$^{\circ,Tot}$**	**ΔS$^{\circ,Rx}$**
273	-202.9	-6.69×10^{-2}	-136.4	-2.02×10^{-1}	-227.7	-7.59×10^{-2}
277	-202.7	-6.66×10^{-2}	-135.7	-2.02×10^{-1}	-227.5	-7.56×10^{-2}
298	-201.7	-6.53×10^{-2}	-131.6	-2.01×10^{-1}	-226.3	-7.43×10^{-2}
303	-201.4	-6.50×10^{-2}	-130.7	-2.01×10^{-1}	-226.0	-7.40×10^{-2}
353	-199.1	-6.26×10^{-2}	-121.1	-2.00×10^{-1}	-223.2	-7.15×10^{-2}

ΔG° and ΔH° = Gibbs free energy and enthalpy in kJ/mol; ΔS° = entropy in kJ/mol˙K; ΔG$^{\circ,Tot}$ = total reaction Gibbs free energy for **prFMN^{im}→prFMN^{ket}** and; ΔS$^{\circ,Rx}$ = reaction entropy of all the elementary processes; T = temperature in K.
